# ZDHHC8 critically regulates seizure susceptibility in epilepsy

**DOI:** 10.1038/s41419-018-0842-0

**Published:** 2018-07-23

**Authors:** Qin Yang, Fangshuo Zheng, Yida Hu, Yi Yang, Yun Li, Guojun Chen, Wei Wang, Miaoqing He, Ruijiao Zhou, Yuanlin Ma, Demei Xu, Xin Tian, Xiaoya Gao, Qing Wang, Xuefeng Wang

**Affiliations:** 1 Department of Neurology, The first Affiliated Hospital of Chongqing Medical University, Chongqing Key Laboratory of Neurology, 1 Youyi Road, Chongqing, 400016 China; 2 Department of Neurology, Zhujiang Hospital of Southern Medical University, Gongye Road 253, Guangzhou, Guangdong Province 510282 China; 3 Center of Epilepsy, Beijing Institute for Brain Disorders, Beijing, 100101 China

## Abstract

Epilepsy is one of the most prevalent and drug-refractory neurological disorders. Zinc finger DHHC-type containing 8 (ZDHHC8) is a putative palmitoyltransferase that is highly expressed in the brain. However, the impact of ZDHHC8 on seizures remains unclear. We aimed to explore the association of ZDHHC8 with epilepsy and investigate its in epileptogenesis in in vivo and in vitro models through behavioral, electrophysiological, and pathological studies. We used kainic acid- and pilocarpine-induced C57BL/6 mice and magnesium-free-induced pyramidal neurons as experimental epileptic models in this study. We first found increased ZDHHC8 expression in the brains of temporal lobe epilepsy (TLE) patients, similar to that observed in chronic epileptic mice, strongly suggesting that ZDHHC8 is correlated with human epilepsy. In the in vitro seizure models, knocking down ZDHHC8 using recombinant adeno-associated virus (rAAV) delayed seizure precipitation and decreased chronic spontaneous recurrent seizures (SRSs) and epileptiform-like discharges, while ZDHHC8 overexpression had the opposite effect. ZDHHC8 levels were consistent with seizure susceptibility in induced mice with SRSs. In an in vitro magnesium-free model, neuronal hyperexcitability and hypersynchrony were reduced in ZDHHC8-knockdown neurons but were increased in ZDHHC8-overexpressing neurons. To further explore the potential mechanisms, we observed that ZDHHC8 had a significant modulatory effect on 2-amino-3-(5-methyl-3-oxo-1,2-oxazol-4-yl) propanoic acid (AMPA) receptor-related excitatory, but not inhibitory, glutamatergic synaptic neurotransmission, further affecting the inward rectification of AMPA currents in acute hippocampal slices in whole-cell recordings. ZDHHC8 facilitated GluA1 trafficking to the neuronal surface in the hippocampus, as shown by immunoprecipitation and Western blotting. These results suggest that ZDHHC8 may promote the generation and propagation of seizures in humans and that knocking down ZDHHC8 might produce anti-epileptogenic effects in drug-resistant epilepsy. Our study provides evidence that may facilitate the development of an alternative approach for the treatment of epilepsy by modulating AMPA/GluA1-mediated neurotransmission.

## Background

Epilepsy is a disabling and drug-refractory neurological disorder characterized by recurrent spontaneous seizures^1^. Approximately 30% of patients do not respond adequately to anti-epileptic drug (AED) treatment and often require lifelong medication^2,3^. Surgical resection of the epileptic focus is an invasive approach and is not an optimal therapeutic option for the majority of resistant patients^3^. Epilepsy has been associated with many pathophysiological mechanisms, including inflammation, gliosis, ion channel dysfunction, and synaptic remodeling^4–6^. However, an imbalance between excitatory and inhibitory neurotransmission that results in hypersynchronous discharge of neurons in a focal area of the brain or throughout the entire brain is the most widely accepted mechanism^1^. Current AEDs primarily target voltage-gated ion channels to reduce neuronal excitability either directly or via synaptic transmission^2,7^. However, AEDs lack efficacy in some patients due to a complex array of brain mechanisms. Thus, a detailed understanding of the regulatory mechanisms responsible for this imbalance is necessary to control seizure susceptibility and develop new therapeutic approaches.

Palmitoylation of proteins is important for neural protein trafficking and function^8,9^, and may provide greater efficacy and tolerability. Zinc finger DHHC-type containing 8 (ZDHHC8), a putative palmitoyltransferase that contains a conserved cysteine-rich signature catalytic (DHHC) domain, is highly expressed in the brain^8^. Previous studies have reported that hemizygous deletion of the ZDHHC8 gene in 22q11 microdeletion carriers may be associated with schizophrenia^10,11^ and neurodevelopmental deficits^12^. ZDHHC8 has also been associated with cerebellar and cortical synaptic plasticity^13,14^. However, no studies investigated the anticonvulsant efficacy of ZDHHC8 on the behavior, electrophysiology, and pathology of chronically epileptic tissue characterized by morphological, molecular, and functional changes.

Therefore, we first examined ZDHHC8 expression and localization in the epileptogenic brain tissues of temporal lobe epilepsy (TLE) subjects and mice. Then, we utilized recombinant adeno-associated virus (rAAV)-mediated knockdown and overexpression of ZDHHC8 in mice to test spontaneous seizures and latent periods in two chronic seizure models. We studied neuronal hyperexcitability and hypersynchrony and their underlying mechanisms in hippocampal slices in an in vitro magnesium-free model. To further explore the mechanisms of ZDHHC8, we monitored its effect on GluA1 trafficking, which might be responsible for seizures. Our results indicate that ZDHHC8 may offer a new strategy for therapeutic interventions in epilepsy.

## Results

### Neuronal localization and expression of ZDHHC8 in TLE patients and epileptic mice

**Fig. 1:**
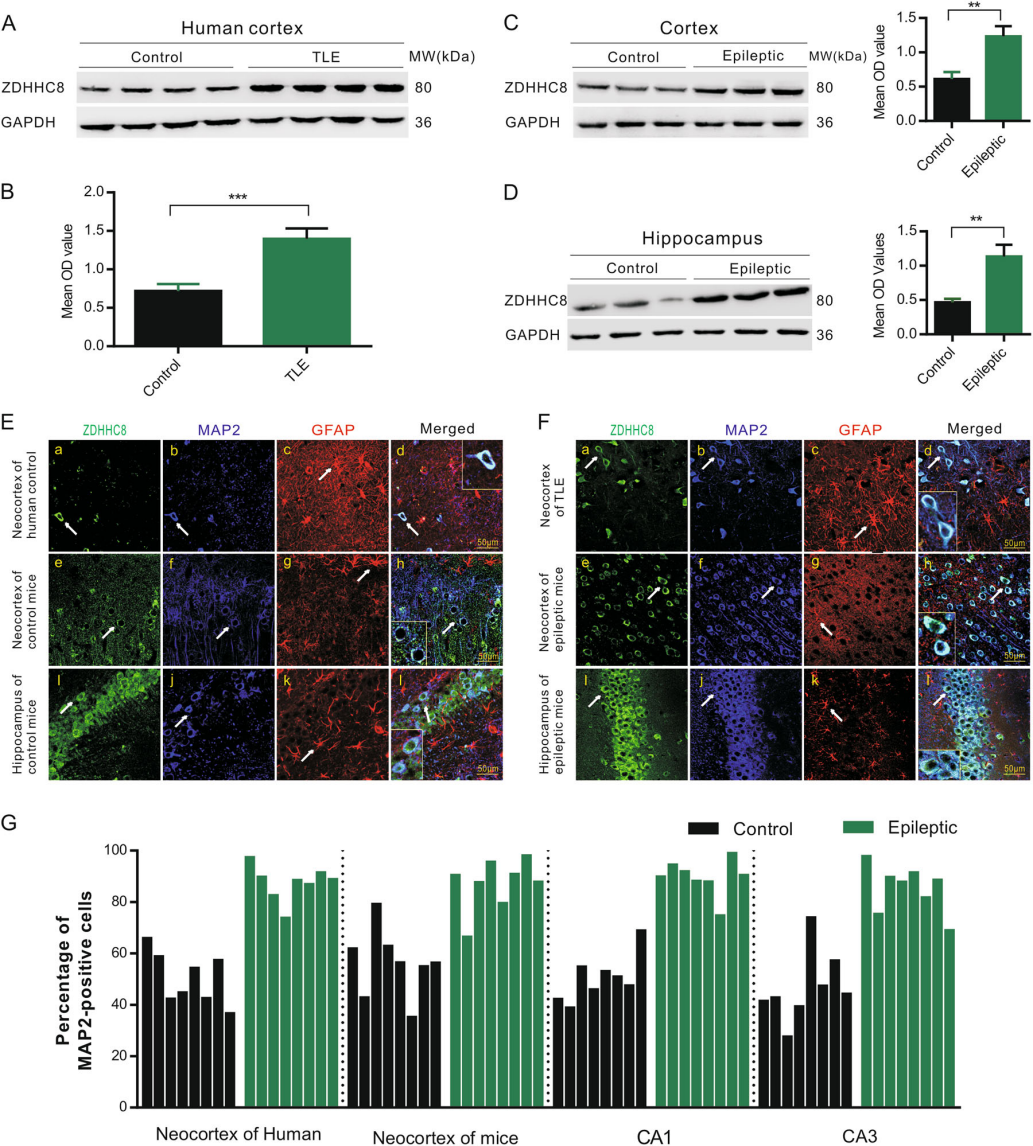
ZDHHC8 upregulation in the brain tissues of epileptic patients and mice. **a, b** ZDHHC8 levels were increased in temporal lobe samples from TLE patients compared with those in the controls. Western blot showing ZDHHC8 levels (*n* = 16 per group, ***P < 0.001 compared to the controls, unpaired *t*-test). **c, d** ZDHHC8 levels were increased in the cortical (**c**) and hippocampal (**d**) lysates of epileptic mice compared with those in the controls (non-epileptic mice) (*n* = 6 per group, **P < 0.01, unpaired *t*-test). **e, f** ZDHHC8 (green) and MAP-2 (blue), but not GFAP (red), co-localized (merged) in the cortex of the temporal lobe samples from the controls, patients with TLE (**a–d**), and epileptic mice (**e–h**) as well as in the hippocampus of the controls and epileptic mice (**i–l**). Arrows indicate positive cells. High-magnification photomicrographs corresponding to specific cells are shown at the bottom. **g** Quantification of ZDHHC8-positive cells co-localized with MAP-2 in the epileptic and control brains in human and mice (*n* = 8)

We first investigated whether in vivo seizure activity affects ZDHHC8 levels. The protein levels of ZDHHC8 in the brain tissues of pharmaco-resistant TLE patients and pilocarpine-induced chronic epileptic mice were tested. Higher levels of ZDHHC8 were detected in TLE specimens than in controls (***P < 0.001, *n* = 16; Fig. 1a, b; Table 1). In pilocarpine-induced epileptic mice, ZDHHC8 levels were also increased in the hippocampus and temporal neocortex (Fig. 1c, d; Fig. S3). In addition, immunofluorescence analysis showed that ZDHHC8 co-localized with the neuronal dendrite-specific marker MAP-2 but not with the astrocytic marker glial fibrillary acidic protein (GFAP) in the controls and both the TLE patients and the epileptic mice (Fig. 1e, f; Fig. S2A). The co-expression of MAP-2 with ZDHHC8-positive cells was strongly increased in the epileptic tissues compared with that in the control tissues, which was consistent with the western blot results (Fig. 1g). These data suggest that ZDHHC8 has similar expression patterns in TLE specimens and chronic epileptic mice and may be associated with human epilepsy.

**Table 1:** Clinical characteristics of TLE and control patients

Subjects (No.)	Gender (M/F)	Age (Y)	Course (Y)	AEDs before the surgery	Resection tissue	Pathological diagnosis
P1	F	22	19	CBZ, OXC, LTG	TNl	NL
P2	F	24	18	VPA, TPM, LTG, OXC	TNl	NL
P3	M	27	10	VPA, LTG, OXC, PB	TNr	NL
P4	M	16	11	VPA, OXC, LVE	TNr	NL
P5	M	20	19	CBZ, OXC, LVE	TNl	G
P6	F	10	10	OXC, TPM, LVE	TNr	NL
P7	M	20	9	OXC, LTG, VPA	TNl	G
P8	M	5	4	LVE, LTG, PB	TNl	NL
P9	F	40	20	CBZ, LTG, TPM	TNl	NL, G
P10	M	31	20	PHT, LVE, LTG	TNl	NL, G
P11	F	23	10	OXC, LTG,CZP	TNl	NL, G
P12	F	25	15	PHT, OXC, VPA	TNl	NL, G
P13	F	36	16	PHT, PB, CBZ, VPA, CZP	TNl	NL, G
P14	M	33	30	VPA, CBZ, CZP	TNl	NL
P15	M	44	20	PHT, PB, CBZ, TPM	TNl	G
P16	M	45	10	TPM, LTG, CBZ	TNl	NL, G
C1	M	54	0	No	TNr	N
C2	F	25	0	No	TNr	N
C3	M	53	0	No	TNl	N
C4	F	42	0	No	TNr	N
C5	F	22	0	No	TNr	N
C6	M	28	0	No	TNl	N
C7	M	15	0	No	TNr	N
C8	F	18	0	No	TNr	N
C9	M	60	0	No	TNl	N
C10	M	23	0	No	TNr	N
C11	M	16	0	No	TNr	N
C12	F	26	0	No	TNr	N
C13	F	27	0	No	TNr	N
C14	F	32	0	No	TNl	N
C15	M	24	0	No	TNl	N
C16	F	35	0	No	TNl	N

*P* patients, *C* control, *F* female, M, male, *Y* years, *AEDs* antiepileptic drugs, *CBZ* carbamazepine, *OXC* oxcarbazepine, *LTG* lamotrigine, *TPM* topiramate, *LVE* levetiracetam, *VPA* valproate, *PB* phenobarbital, *PHT* phenytoin, *CZP* clonazepam, *TN* temporal neocortex, *l* left, *r* right, *NL* neuron loss, *G* Gliosis, *N* normal.

### In vivo effect of ZDHHC8 on seizure susceptibility in animal models

To determine if changes in the levels of ZDHHC8 had any effects on seizure susceptibility, we generated a kainic acid (KA)-induced chronic seizure model. The KA model appears to be highly isomorphic to TLE^15^. The experimental mice were used for analysis 3 weeks after rAAV vector injection (Fig. S1A-S1D). Hematoxylin–eosin (HE) staining indicated that the brains of the mice injected with rAAV had normal gross anatomy (Fig. S1E).

The activity of the observed phenotype might be changed in the status epilepticus (SE), thereby impacting the development of chronic epilepsy^16,17^. We first sought to confirm whether ZDHHC8 affects acute seizures in KA-induced SE. Interestingly, no differences in behavioral (Fig. S4A-S4D) and electrographic (Fig. S4E-S4H) seizures during SE were detectable in rAAV-ZDHHC8-sh-injected and rAAV-ZDHHC8-injected mice compared with control mice. Moreover, analyses of ZDHHC8 protein levels revealed that they were not altered between the SE and non-SE groups (Fig. S6A). These results provide evidence that ZDHHC8 does not affect KA-induced SE.

**Fig. 2:**
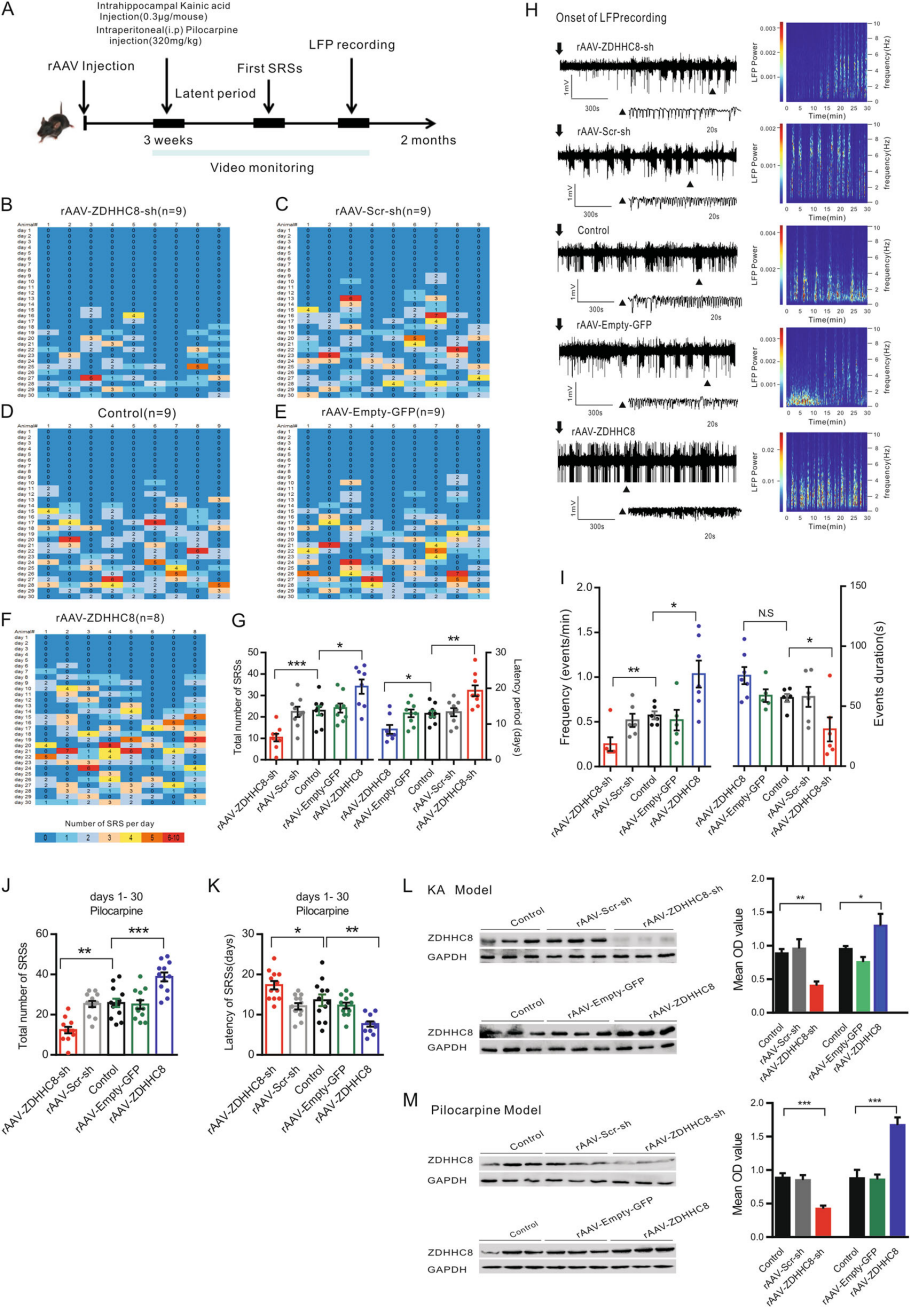
ZDHHC8 regulates seizure susceptibility in chronic seizure models in vivo. **a** Schematic of the experimental protocol. Mice were administered rAAV via intracerebroventricular (i.c.v.) injection after 3 weeks. These mice were then intrahippocampally injected with KA (0.3 µg/mouse) or intraperitoneally (i.p.) injected with pilocarpine (320 mg/kg). **b–f** Heat map showing the number of SRSs detected each day during days 1–30 after SE induction in the KA-induced seizure model. **g** Quantification of ZDHHC8 effects on KA-induced SRSs (left) and latency period (right). **h** Representative LFP tracings from KA-induced epileptic mice with SRSs. EEG seizures were recorded in C57BL/6 mice with stable baseline spontaneous seizures for 2 h before ending the experiment. **i** Quantification of ZDHHC8 effects on the frequency and duration of epileptiform-like discharge events in LFP (rAAV-ZDHHC8-sh, *n* = 6; rAAV-Scr-sh, *n* = 6; Control, *n* = 5; rAAV-Empty-GFP, *n* = 6; and rAAV-ZDHHC8, *n* = 7). **j,** **k** Quantification of ZDHHC8 effects on pilocarpine-induced SRSs (**j**) and latency period (**k**) during days 1–30 after SE induction (rAAV-ZDHHC8-sh, *n* = 12; rAAV-Scr-sh, *n* = 13; Control, *n* = 13; rAAV-Empty-GFP, *n* = 12; and rAAV-ZDHHC8, *n* = 12). **l, m** Representative western blots of hippocampal lysates. Quantification of ZDHHC8 protein levels in the hippocampus of C57BL/6 mice with stable SRSs in KA-/ (**l**) and pilocarpine-induced models (**m**) (*n* = 5). Error bars represent the mean ± SEM. *P < 0.05, **P < 0.01, and ***P < 0.001; one-way ANOVA

We next sought to verify whether ZDHHC8 changed the development of chronic, spontaneous recurrent seizures (SRSs). SRSs were detected using continuous video monitoring after SE (Fig. 2a, f). SE spontaneously remitted within 24 h of KA administration, and the first spontaneous seizures were generally observed between 5 and 30 days later, with an average of 14 days after KA injection^15,18^. Consistent with the normal course of epilepsy in this model, the period before the first spontaneous seizure after SE (latency) averaged 13 days in rAAV-Scr-sh, rAAV-Empty-GFP or control mice, whereas rAAV-ZDHHC8-sh-injected mice were epileptic by day 19.33 ± 1.57 (*P* = 0.0011, Fig. 2g, right). Fewer SRSs were observed in the rAAV-ZDHHC8-sh (10.33 ± 1.76) animals compared with the rAAV-Scr-sh (23.33 ± 2.82) and control (22.33 ± 2.40) animals during the 1–30 days immediately after SE (*P* = 0.0004, Fig. 2g, left), indicating that rAAV-ZDHHC8-sh treatment attenuated SRSs recurrence and retarded seizure precipitation. In contrast, ZDHHC8 upregulation increased the number of SRSs and shortened the latency (Fig. 2g).

To measure changes in epileptiform activity, we recorded local field potentials (LFPs) in the KA model mice with stable baseline SRSs (Fig. 2h). LFP analysis revealed that the frequency and duration of high-amplitude, high-frequency discharges, called epileptiform-like discharges, which are associated with damage-causing pathologic activity^17,19^, were reduced in the rAAV-ZDHHC8-sh-injected mice (frequency, *P* = 0.0084; duration, *P* = 0.0429; Fig. 2i), as were the energy spectra. This effect was qualitatively similar to the seizure suppression observed in the animals. To further assess this hypothesis, we performed the opposite study by injecting rAAV-ZDHHC8 into mice treated with KA; however, the duration of epileptiform-like discharges showed no change (Fig. 2i). Pilocarpine-induced chronic seizures are similar to those observed in human TLE patients^20^. We also measured ZDHHC8-mediated seizure susceptibility in this model, as described in our previous work^21^, and behavioral seizures were consistent with the KA results (Fig. 2j, k; Fig. S5; Fig. S6B; Fig. S7).

According to these results, seizure degree and the level of ZDHHC8 expression appeared to be related. Finally, we performed western blots in each group of epileptic mice an average of 9 weeks after SE (Fig. 2l, m). The rAAV-ZDHHC8-sh-injected mice displayed lower ZDHHC8 expression compared with the controls, consistent with the spontaneous seizures and latency findings. Similarly, the higher level of ZDHHC8 in the rAAV-ZDHHC8-injected mice reflected the appearance of multiple spontaneous seizures. Therefore, ZDHHC8 expression was linked with seizure susceptibility, further confirming the precisely targeted vector injection. Overall, ZDHHC8 regulated seizure susceptibility in different chronic seizure models related to TLE.

### In vitro effect of ZDHHC8 on neuronal hyperexcitability and hypersynchrony in an Mg^2+^-free seizure model

**Fig. 3:**
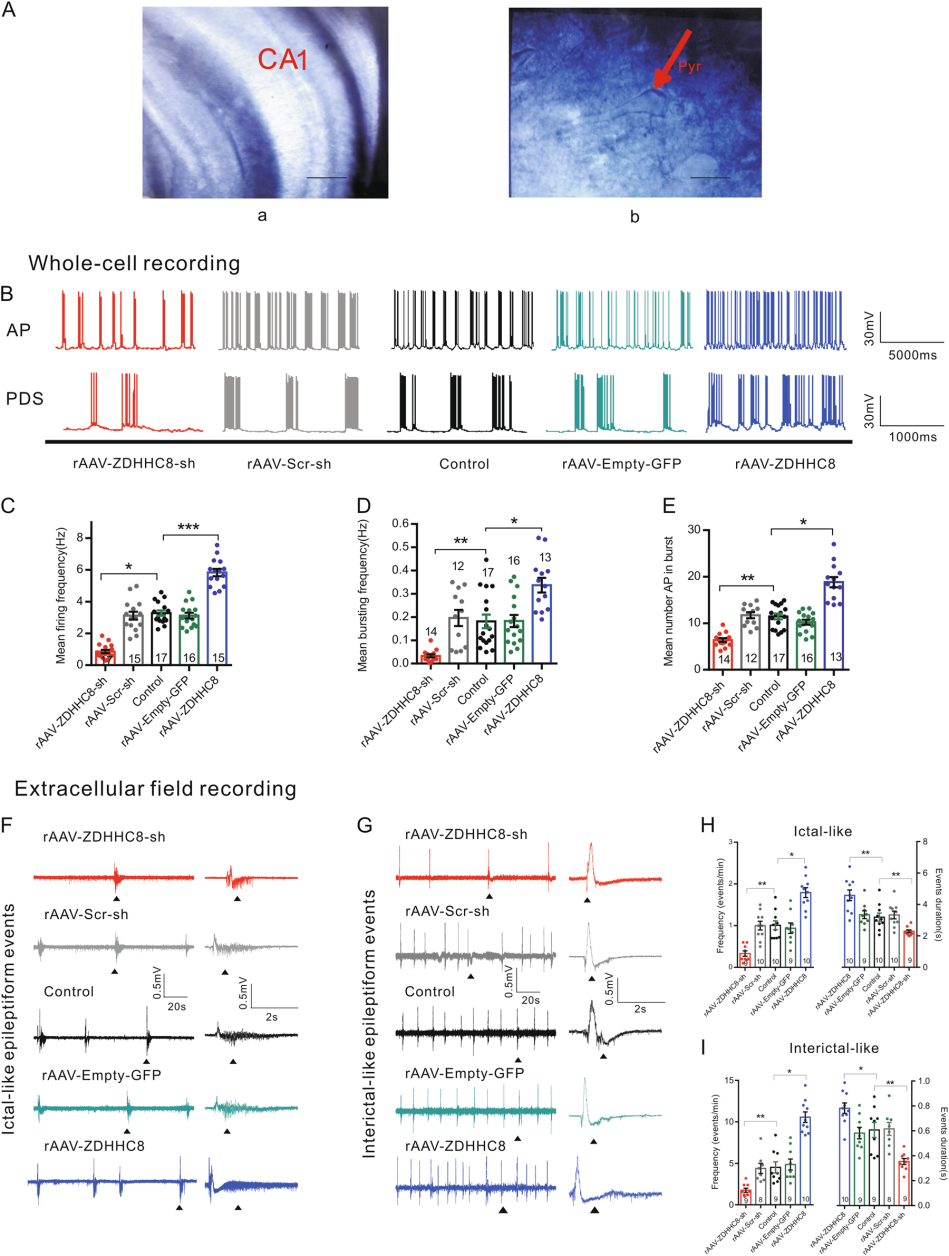
In vitro Mg2+-free model whole-cell and extracellular field recordings showing the effect of ZDHHC8 on neuronal hyperexcitability in hippocampal slices. **a** Identification of pyramidal neurons in the CA1 region of hippocampal slices. (**a**) Representative image of the CA1 region; scale bar, 100 µm. (**b**) Pyramidal neurons (red arrowheads) have a typical soma (cell body) shaped like a teardrop or a rounded pyramid and one projecting axon to which the dendrites bind in a thick band called the stratum radiatum; scale bar, 10 µm. **b** APs and PDSs of a pyramidal neuron triggered by the omission of Mg^2+^ from the ACSF by whole-cell patch-clamp recordings (holding the resting membrane potential constant) in rAAV-treated and control mice. Top: trace of a cell with spontaneous APs. Bottom: trace of a cell with PDSs. **c** Quantification of the mean spontaneous firing frequency (*n* = 4 mice per group). **d, e** Quantification of the frequency of PDSs (**d**) and the average number of APs in PDSs (**e**) (*n* = 4 mice per group). **f–i** Epileptiform-like activity elicited in hippocampal slices by removing extracellular Mg^2+^ and adding 8.5 mM K^+^ to the perfusate in the in vitro model. Representative ictal- (**f**) and interictal-like (**g**) epileptiform events elicited in the CA1 region. Average frequencies (events/min) (left) and event durations (right) of ictal-like (**h**) and interictal-like events (**i**) (unit: slices, *n* = 4 mice per group). All data represent the mean ± SEM. Statistical significance (*P < 0.05, **P < 0.01, and ***P < 0.001) was evaluated by one-way ANOVA

Neuronal hyperexcitability and hypersynchrony, which are the key characteristics of epilepsy, have been simulated in many in vitro seizure models^22^. We determined whether the ZDHHC8-mediated promotion of spontaneous seizures is dependent on neuronal hyperexcitability and hypersynchrony by downregulating or upregulating ZDHHC8 and performing whole-cell recording in acute hippocampal slices in an in vitro Mg^2+^-free seizure model (Fig. 3a).

Recurrent action potentials (APs) are primarily responsible for neuronal hyperexcitability in epileptic conditions^1,23^. In our study, the frequency of APs from CA1 pyramidal was significantly reduced in the rAAV-ZDHHC8-sh-injected slices (0.85 ± 0.11 Hz) compared with the rAAV-Scr-sh (3.12 ± 0.25 Hz) and control (3.28 ± 0.16 Hz, *P* = 0.043) slices, whereas this frequency was increased in the rAAV-ZDHHC8-injected slices (5.83 ± 0.23 Hz, *P* = 0.0016, Fig. 3b, c). The paroxysmal depolarization shift (PDS) represents single neuronal hypersynchrony, which is initiated from neuronal depolarizations eliciting trains of APs (Fig. 3b) and is correlated with interictal spikes in the EEGs of epileptic patients^24^. The rAAV-ZDHHC8-sh-injected slices (0.03 ± 0.006 Hz) showed a reduction in the burst (PDS) frequency compared with the rAAV-Scr-sh (0.20 ± 0.03 Hz) and control (0.18 ± 0.03 Hz, *P* = 0.001, Fig. 3d) slices; these slices also showed a reduction in the average number of APs in PDSs (*P* = 0.007, Fig. 3e). To further verify these results, we analyzed rAAV-ZDHHC8-injected slices (Fig. 3d, e), which exhibited the opposite results. These findings demonstrate that neuronal hyperexcitability and hypersynchrony are significantly reduced in ZDHHC8-knockdown pyramidal neurons but are increased in ZDHHC8-overexpressing neurons.

Next, to further elucidate the role of ZDHHC8 in the pathological synchronization of neuronal networks, we assessed epileptiform activity (synchronous activity) in multiple neurons of the CA1 region using extracellularly recorded field potentials (Fig. 3f, g). To obtain more manifest epileptiform discharges, we exposed all the slices to a high-potassium and Mg^2+^-free solution. Compared with the control and rAAV-Scr-sh slices, the rAAV-ZDHHC8-sh-treated slices showed decreased frequencies and event durations for ictal-like and interictal-like events, while these parameters were increased in the rAAV-ZDHHC8-treated slices (Fig. 3h, i), indicating that ZDHHC8 is sufficient to induce the abnormal excitability of epileptic neural networks in vitro.

### ZDHHC8 affects postsynaptic neurotransmission

**Fig. 4:**
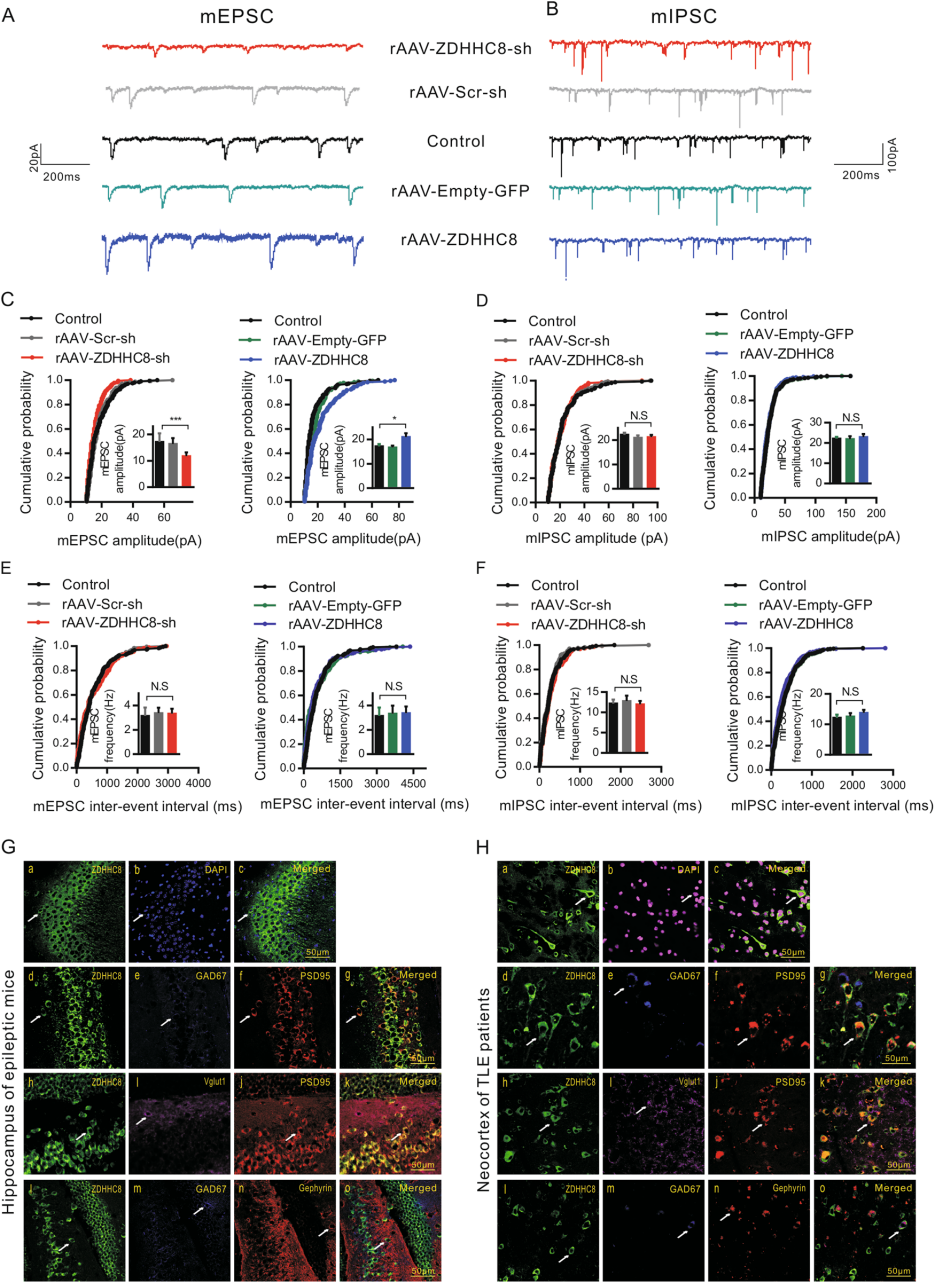
ZDHHC8 may alter the postsynaptic glutamatergic receptor response probability. **a, b** Representative traces showing mEPSCs (**a**) and mIPSCs (**b**) recorded from CA1 pyramidal cells. **c, d** Cumulative probability plots of mEPSC (**c**) and mIPSC amplitudes (**d**). Insets show bar graphs of the amplitudes (mEPSCs: rAAV-ZDHHC8-sh, 18 cells; rAAV-Scr-sh, 12 cells; Control, 12 cells; rAAV-Empty-GFP, 12 cells; and rAAV-ZDHHC8, 18 cells; *n* = 4 mice per group; left, ***P < 0.001, right, *P < 0.05); mIPSCs: rAAV-ZDHHC8-sh, 18 cells; rAAV-Scr-sh, 15 cells; Control, 20 cells; rAAV-Empty-GFP, 18 cells; and rAAV-ZDHHC8, 18 cells; *n* = 4 mice per group). **e**, **f** Cumulative probability plots of mEPSC (**e**) and mIPSC (**f**) frequencies (measured by inter-event intervals). Insets display bar graphs of frequencies (the numbers of cells in **c**, **d**). Data represent the mean ± SEM. Statistical significance was evaluated with the KS-test (cumulative probability plots) and one-way ANOVA (*P < 0.05, ***P < 0.001; N.S. represents no significance). **g**, **h** Localization of ZDHHC8 in the hippocampus of chronic epileptic mice (**g**) and in the neocortex of TLE patients (**h**). **a–c** ZDHHC8 was expressed (green) in the cell membrane but not the cytoplasm. **d–g** ZDHHC8 expression (green) and PSD95 (red) were co-localized, indicating that ZDHHC8 was located at excitatory synapses. **h–k** ZDHHC8 expression (green) and VGLUT1 (purple) were not co-localized, indicating that ZDHHC8 was located in postsynaptic membranes. **l–o** ZDHHC8 expression (green), GAD67 (blue), and Gephyrin (red) were not co-localized, indicating that ZDHHC8 was not located in inhibitory synapses; scale bar, 50 μm. The white arrows in all the images indicate positive neurons

The observed pathological excitability suggests that the disturbances in synaptic transmission in pyramidal neurons are related to changes in ZDHHC8 regulation. We measured glutamatergic synaptic transmission and GABAergic synaptic transmission in the CA1 pyramidal neurons of hippocampal slices via whole-cell recordings with Mg^2+^-free artificial cerebrospinal fluid (ACSF) (Fig. 4a, b). We observed a significantly lower mean amplitude of miniature excitatory postsynaptic currents (mEPSCs) (*P* = 0.0006, Fig. 4c, left) but not miniature inhibitory postsynaptic currents (mIPSCs) (*P* = 0.4896, Fig. 4d, left), in slices from the ZDHHC8-knockdown mice compared with those from the control and rAAV-Scr-sh mice. The cumulative probability curve also indicated a leftward shift toward smaller amplitudes (Fig. 4c, left). We further investigated this finding and found that the mEPSC amplitude of the ZDHHC8-overexpressing mice was increased (*P* = 0.0212, Fig. 4c, right) without a change in the mIPSC amplitude (*P* = 0.5047, Fig. 4d, right). The cumulative probability curve exhibited a rightward shift toward larger amplitudes (Fig. 4e, right). Additionally, no change in the mEPSC and mIPSC frequencies was observed (Fig. 4e, f), suggesting that ZDHHC8 did not alter the quantal size of the presynaptic transmitters. This selective deficit in mEPSC amplitude suggests that ZDHHC8 mutations may alter postsynaptic glutamatergic receptor response probabilities, whereas inhibitory events are not altered.

This hypothesis was further verified by immunofluorescence staining, which showed that ZDHHC8 was distributed in the membranes of neurons in both epileptic and control tissues (Fig. 4g, h; Fig. S2B, a-c), consistent with previous reports that ZDHHC8 is a transmembrane protein^8^. Consistent with the mEPSC findings, we observed ZDHHC8 to co-localize with PSD95-positive neurons but not with GAD67 (an enzyme that produces the inhibitory neurotransmitter GABA)- (Fig. 4g, h, d–g; Fig. S2B, d-g) or Gephyrin (a major scaffolding protein at inhibitory synapses)-positive neurons (Fig. 4g, h, i–o; Fig. S2B, i-o). Nevertheless, ZDHHC8 did not co-localize with the presynaptic marker vesicular glutamate transporter 1 (Vglut1), but it localized with PSD95 in excitatory pyramidal neurons (Fig. 4g–k; Fig. S2B, h-k), suggesting that ZDHHC8 is present in individual postsynaptic neurons. Consequently, these experiments demonstrated that ZDHHC8-mediated neurotransmission increases excitatory, but not inhibitory, synaptic input through a postsynaptic receptor-dependent mechanism.

### ZDHHC8 is involved in neurotransmission through GluA2-lacking calcium-permeable AMPARs in the hippocampus

**Fig. 5:**
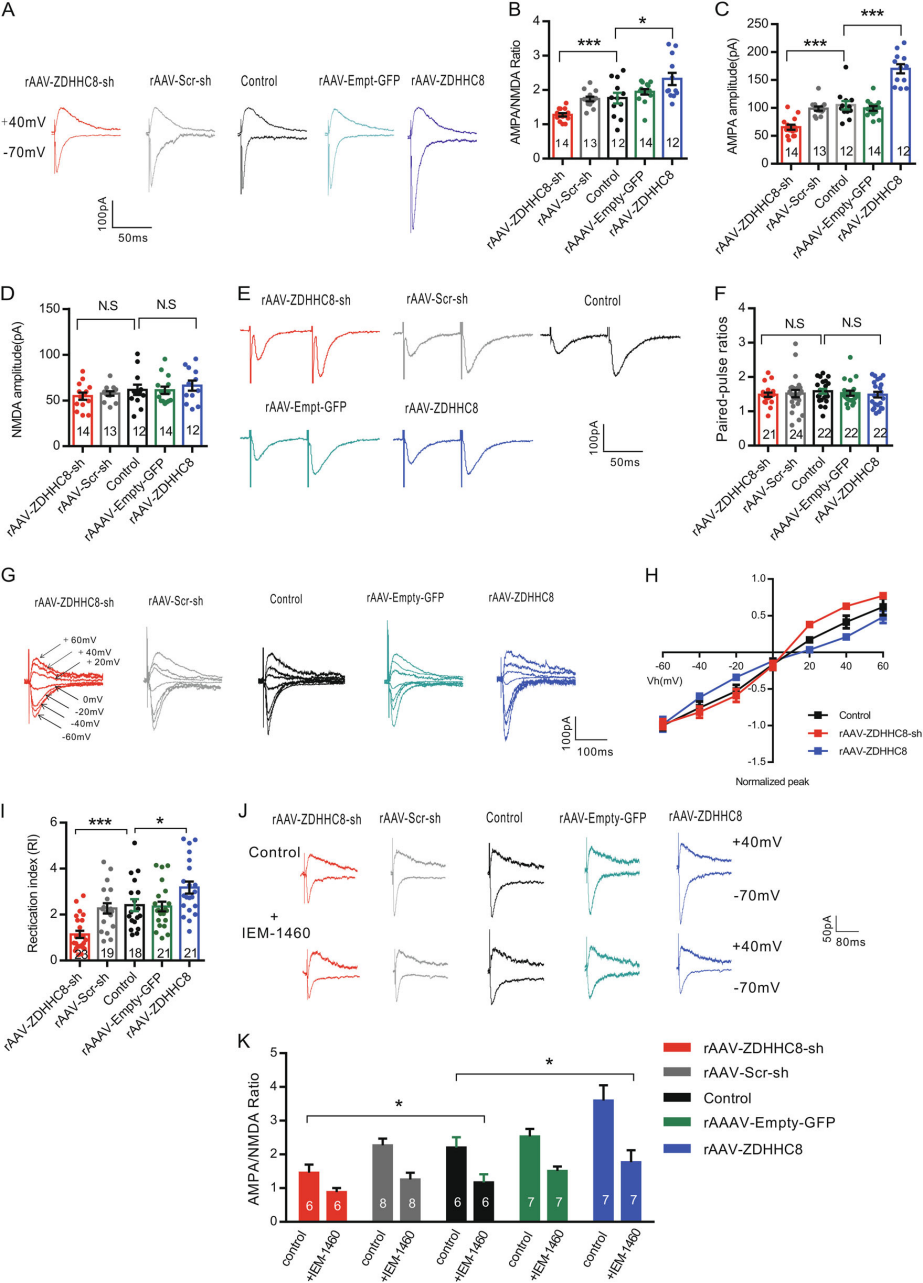
ZDHHC8 regulates postsynaptic AMPA receptor function in the Mg^2+^-free model in vitro. **a** Representative traces showing evoked AMPARs (bottom; holding at −70 mV) and NMDAR EPSCs (top; holding at +40 mV) recorded from CA1 pyramidal cells in hippocampal slices. **b** Bar graphs showing the ratio of AMPAR/NMDAR EPSCs (*n* = 4 mice per group; *P < 0.05, ***P < 0.01). **c, d** Summary of the effects of ZDHHC8 on absolute peak current amplitudes mediated by AMPARs or NMDARs. NMDAR-mediated responses were measured at 50 ms post-stimulus (the numbers of cells in **b**, *P < 0.05, ***P < 0.001; N.S. represents no significance). **e** Representative sample traces of paired-pulse facilitation measurements obtained with a 50-ms inter-stimulus. **f** Bar graphs showing the PPR (*n* = 4 mice per group; *P* > 0.05). **g–i** Current–voltage (*I*–*V*) relationships were derived for AMPAR-mediated currents from CA1 pyramidal cells. **g** Representative traces of AMPA currents were recorded at various holding potentials (−60, −40, −20, 0, +20, +40, and +60 mV). **h** *I*–*V* curves of AMPA currents. All AMPAR EPSC values were normalized at −60 mV as a function of holding potential (mV), showing the inward rectification at positive holding potentials in ordinate. **i** Bar plot of the rectification index (RI) (RI was calculated as the AMPAR-mediated current response at −60 mV/+40 mV; *n* = 4 mice per group; *P < 0.05, ***P < 0.001; one-way ANOVA). **j, k** Representative traces (**j**) and bar plot (**k**) of AMPAR/NMDAR EPSCs (top) before and after IEM-1460 (50 µM) application (bottom). The data represent the mean ± SEM (*P < 0.05; two-way ANOVA with Bonferroni’s post hoc test)

Measurements of glutamatergic synaptic strength were performed as previously described^25^ and defined the amplitudes of AMPA receptor-mediated EPSCs relative to the amplitudes of NMDA receptor-mediated EPSCs as the so-called AMPA/NMDA ratio. To further determine whether a ZDHHC8 mutation causes a deficit in excitatory synaptic transmission, we measured excitatory synaptic strength by assessing the AMPA/NMDA ratio in the CA1 region of hippocampal slices, as shown in Fig. 5a. In this assay, rAAV-ZDHHC8-sh-treated slices produced a significant decrease in the AMPA/NMDA ratio, while an increase was observed in the rAAV-ZDHHC8-treated slices (Fig. 5b). These results were consistent with the mEPSC findings, which indicated that impairment in synaptic strength occurs through a postsynaptic receptor-dependent neurotransmission mechanism in ZDHHC8-mutant mice. We investigated whether this abnormality in synaptic strength was due to changes in AMPA receptor (AMPAR) function and/or NMDA receptor (NMDAR) function. The results showed that AMPAR-mediated current amplitudes were significantly reduced compared with those from the control and rAAV-Scr-sh mice in the rAAV-ZDHHC8-sh slices, in contrast to the results from the rAAV-ZDHHC8 slices (Fig. 5c). ZDHHC8 had no effect on the amplitudes of NMDAR-mediated currents (Fig. 5d). Finally, we studied the paired-pulse ratio (PPR; Fig. 5e), a sensitive index of presynaptic release probability, which did not differ between the groups (Fig. 5f). Therefore, these results suggest that ZDHHC8 affected the postsynaptic molecular machinery involved in AMPA-mediated excitatory transmission.

AMPAR function is largely dependent on subunit composition^26^. To further elucidate the processes involved in AMPAR function, we calculated rectification indexes (RIs) and compared current–voltage (*I*–*V*) relationships for AMPAR-mediated currents (Fig. 5g). Thismethod has previously been used to show that adding 100 μM spermine, a functional inhibitor of receptors containing GluA1 subunits and lacking GluA2 subunits, to the recording electrode generates a characteristic inward-rectifying *I*–*V* relationship at positive potentials^25,27^. The results showed a decrease in inward-rectifying AMPA currents in slices from the rAAV-ZDHHC8-sh-treated mice and an increase in slices from the rAAV-ZDHHC8-treated mice compared with the results of the AAV-Empty-GFP and control slices (Fig. 5h). The ratio of peak current amplitude at −60 mV to that at +40 mV mirrored these data (Fig. 5i). To further test this possibility, we evaluated the AMPA/NMDA ratio before perfusing slices with IEM-1460 (Fig. 5j) and found that the AMPA/NMDA ratio markedly deceased in all the groups (Fig. 5k). Therefore, these results suggest that AMPA receptors containing GluA1, but not GluA2, subunits contribute to the functional responses in glutamatergic transmission induced by ZDHHC8 knockdown or overexpression, which results in hyper-excitability and eventually seizure activity.

### ZDHHC8 facilitates GluA1 trafficking to the neuronal surface in the hippocampus

**Fig. 6:**
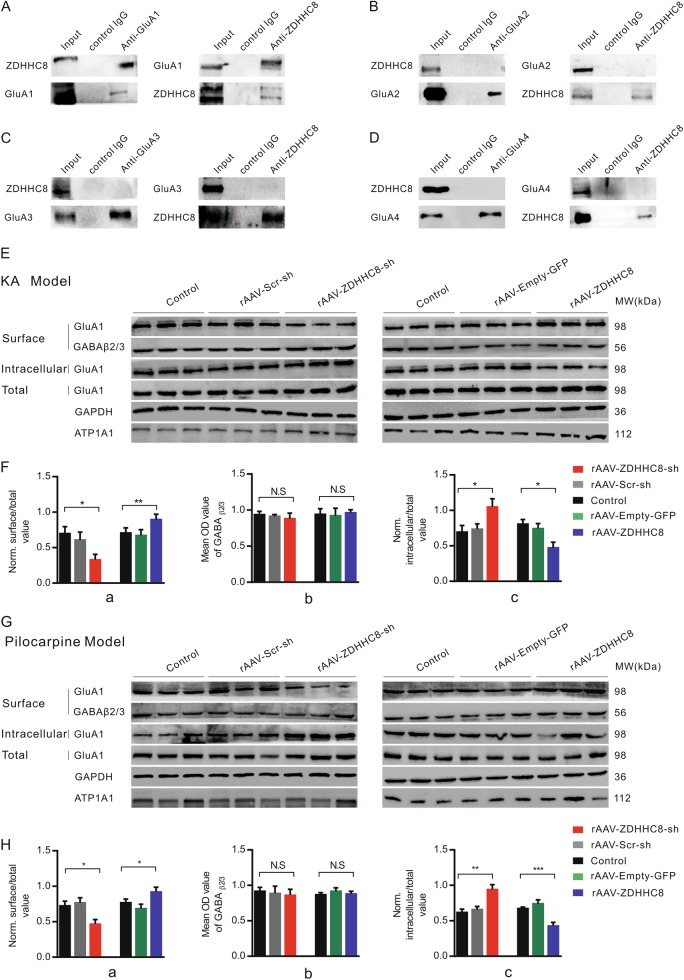
ZDHHC8 interacts with GluA1 and regulates GluA1 trafficking to the neuronal surface in the hippocampus. **a–d** Immunoprecipitation of KA-induced epileptic mice hippocampal lysates. ZDHHC8 co-immunoprecipitates with GluA1 (**a**), GluA2 (**b**), GluA3 (**c**), and GluA 4 (**d**). ZDHHC8 was mainly bound to GluA1 (**a**). **e–h** Western blot analysis of the cell expression of GluA1 in hippocampal tissues from rAAV-ZDHHC8-sh, rAAV-Scr-sh, Control, rAAV-Empty-GFP, and rAAV-ZDHHC8 mice in the KA- (**e**, **f**) and pilocarpine-induced (**g**, **h**) chronic seizure models. **f**(**a**), **f**(**b**); **h**(**a**), **h**(**b**) Bar graph showing that the amount of cell-surface GluA1 decreased significantly in rAAV-ZDHHC8-sh tissues and increased in rAAV-ZDHHC8-overexpressing tissues, whereas that of GABAA β2/3 was unchanged. **f(c); h(c)** Intracellular GluA1 expression behaved opposite to that of GluA1 cell-surface expression. The levels were normalized to the total protein for each receptor. Error bars indicate the mean ± SEM, *n* = 5 independent experiments for each group, *P < 0.05, **P < 0.01, and ***P < 0.001; one-way ANOVA

To further investigate the subunit-dependence of the ZDHHC8 regulation of AMPA receptors, we performed co-immunoprecipitation assays of all AMPAR subunits in hippocampal tissues from chronic seizure mice. Consistent with the *I*–*V* relationship findings, ZDHHC8 only interacted with the GluA1 protein (Fig. 6a–d), indicating that ZDHHC8 achieves its excitatory synaptic output via GluA1 subunits.

Because AMPAR trafficking is highly dynamic at the neuronal surface, we examined surface protein expression levels (Fig. 6e–h). Cell-surface GluA1 expression was significantly reduced in ZDHHC8-knockdown neurons from pilocarpine-induced and KA-induced chronic seizure mice, while GABAAR β2/3 expression was unchanged (Fig. 6f(a, b) and 6h(a, b)). Similarly, we investigated GluA1 in the intracellular fraction using the remaining cell lysate after the cell-surface fraction was removed and found that GluA1 levels were increased compared with those in the control and rAAV-Scr-sh neurons (Fig. 6f(c); 6h(c)). In addition, total GluA1 levels did not significantly change among the groups (Fig. 6e–g). These data suggest that ZDHHC8 knockdown does not affect overall GluA1 expression but instead alters GluA1 subcellular localization. Indeed, we found that the cell-surface expression of GluA1 was increased in ZDHHC8-overexpressing neurons (Fig. 6e–h). These experiments demonstrated that ZDHHC8 participates in GluA1 trafficking by contributing to AMPAR-mediated functional responses and seizure susceptibility.

## Discussion

The present study identifies a previously unknown role for ZDHHC8 in the generation and recurrence of seizures. We utilized two in vivo seizure models based on KA- or pilocarpine-induced SRS and an in vitro Mg^2+^-free seizure model. We also detected high levels of ZDHHC8 expression in the brain tissues of both TLE patients and epileptic mice. Furthermore, electrophysiological studies showed a reduction in only the mEPSC amplitude in ZDHHC8-knockdown neurons, suggesting a change in excitatory, but not inhibitory, neurotransmission. Both the AMPA/NMDA ratio and the PPR indicated that ZDHHC8 was involved in postsynaptic APMA receptor function. In addition, ZDHHC8 regulated GluA1 trafficking to the neuronal surface in the hippocampus, which is responsible for hyperexcitability and eventual seizure activity.

In experimental models of chronic epilepsy and in patients with TLE, the brain displays several abnormalities in the form of morphological and functional synaptic rearrangements^28^. An association between seizures and changes in protein expression has been suggested by recent profiling work^29–31^. Our experiments revealed increased ZDHHC8 expression in the neocortical tissue of TLE subjects, similar to that observed in the hippocampus and adjacent cortex of chronic seizure model mice, suggesting that in vivo regulation of ZDHHC8 is coupled to epileptic or pathogenic brain activity and that ZDHHC8 plays a role in human epilepsy. We were unable to compare hippocampal ZDHHC8 expression with that in the neocortex (control) in TLE patients due to practical and ethical concerns. However, the animal study provides direct evidence that seizure activity leads to increased ZDHHC8 in the hippocampus and cortex.

Although it is not clear that ZDHHC8 participates in the chain of events leading to the precipitation and recurrence of seizures, our animal studies indicated that behavioral seizures seldom recurred in the rAAV-ZDHHC8-sh-treated animals but were higher in the rAAV-ZDHHC8-treated mice, whereas epilepsy developed normally in the control and rAAV-Empty-GFP-treated mice. These findings are consistent with previous reports that AEDs were effective in animal models^15,20^, suggesting that ZDHHC8 inhibition can interrupt epileptogenesis or prevent epileptic seizures from occurring due to a prolonged anti-epileptogenic effect. Although the two chronic seizure models yielded similar results in terms of latency to SRSs, behavioral symptoms, and average total numbers of SRSs, these outcomes were induced by different methods. Intrahippocampal administration of KA was used to investigate the effect of ZDHHC8 on seizures and epileptiform activities in circumscribed pathological networks and was propagated throughout the brain, whereas systemic injection of pilocarpine was employed to study susceptibility to seizures under conditions of a more widespread epileptic disease. The changes in the frequency of spontaneous epileptiform discharge in LFPs in animals also supported the behavioral results. However, the mean duration of epileptiform events did not markedly differ between the ZDHHC8-overexpressing mice and the control mice. These effects might be related to the degree of activation, the density of active neurons in the area, and the temporal pattern of the input^32^, though the exact mechanisms underlying these differences are currently unknown. The hyperexcitability of epileptic neurons and abnormal hypersynchronous discharge of neuronal networks result in epileptic seizures. Hippocampus is prone to the generation of epileptiform activity and seizures^1^. In our in vitro Mg^2+^-free seizure model, electrophysiological data clearly showed that epileptiform activity in the firing of single neurons and networks in the CA1 area of hippocampal slices was inhibited by ZDHHC8 knockdown and increased by ZDHHC8 overexpression. Thus, ZDHHC8 may represent a major therapeutic target in epilepsy.

The mechanism by which ZDHHC8 regulates seizure susceptibility is unknown, but our study suggests the intriguing possibility that the effect occurs via changes to AMPA receptors. We determined the effect of ZDHHC8 on synaptic transmission in the CA1 area of the hippocampus and found that ZDHHC8 regulated mEPSC amplitude in epileptic environments, in a manner consistent with a postsynaptic effect, similar to previous reports that ZDHHC8 is associated with excitatory receptors^8,12,13^. Moreover, ZDHHC8 had no appreciable effect on inhibitory transmission. Our immunofluorescence labeling also supported this hypothesis. The alternations in the AMPAR-to-NMDAR EPSC ratio elicited by ZDHHC8 reflect the regulation of AMPAR-mediated excitatory synaptic transmission. These findings are consistent with previous reports that AMPA receptors are targets for seizure control and mediate generation and propagation of epileptic activity^33,34^. AMPARs are distributed in areas of the cerebral cortex, amygdala, thalamus, and hippocampus, which are all relevant to epilepsy^35^. Furthermore, blocking AMPARs can inhibit seizures in various in vitro and in vivo epilepsy models^33,34^. Here, we showed that AMPA receptor antagonism by ZDHHC8 knockdown is sufficient to explain its antiseizure effect. These results were confirmed using perampanel, a novel AMPA receptor antagonist that has already been approved to treat refractory epilepsy^36^. Thus, our results showing that ZDHHC8 knockdown reduces the magnitude of fast glutamatergic signaling in the hippocampus are consistent with previous reports that AMPA receptors are a recognized target for antiseizure therapies^37^. Conversely, ZDHHC8 overexpression can promote the onset of epileptic seizures.

Notably, AMPAR subunits are increased in association with chronic temporal lobe seizures, and alterations in receptor subunit composition probably contribute to neuronal hyperexcitability, excitatory neurotransmission and seizure susceptibility^7,38,39^. Our experiments revealed that GluA1 protein levels decreased in the rAAV-ZDHHC8-sh-treated epileptic mice and increased in the rAAV-ZDHHC8-treated epileptic mice, indicating that ZDHHC8 regulates seizure susceptibility through GluA1 subunits during postsynaptic activation and seizure propagation. However, AMPAR-mediated excitatory synaptic transmission is highly dynamic in the brain. Many proteins interact with AMPAR subunits and affect their synaptic localization. AMPARs are also continuously transported in and out of synapses by membrane trafficking, which involves insertion and internalization via specific vesicles^40^. We found that cell-surface GluA1 expression was decreased and cytosolic expression was increased in ZDHHC8-knockdown mice, whereas total protein levels did not change, indicating increased GluA1 endocytosis or impaired GluA1 trafficking from intracellular pools to the cell surface. Therefore, ZDHHC8 may regulate the trafficking of GluA1 subunits, resulting in seizure activity.

In summary, to our knowledge, this is the first study to demonstrate increased ZDHHC8 expression in brain specimens from TLE subjects. These subjects are highly prone to seizure precipitation, suggesting that the ZDHHC8 may play a key role in human epilepsy. Although reduced GluA1 AMPAR trafficking to the plasma membrane by ZDHHC8 knockdown as the mechanism for the seizure suppressant effects is a compelling speculation, few studies have indicated that ZDHHC8 knockdown can be used as an optimal target for anti-epileptic treatment or that ZDHHC8 regulates AMPAR trafficking in seizures. Therefore, our findings may represent a partial compensatory mechanism in seizure susceptibility. Our study provides evidence that may aid in the development of alternative approaches to treat epilepsy through the modulation of AMPA/GluA1-mediated neurotransmission.

## Materials and methods

### Human samples

All human temporal neocortical and mouse samples were collected as described in our previous study^29,30^. Sixteen patients (9 males and 7 females; mean age 26.31 ± 2.85 years (5–45 years); mean disease course 15.06 ± 1.60 (4–30 years)) who had undergone surgical resection of the temporal lobe were randomly chosen from 400 specimens in our epileptic brain tissue bank. For comparison, we randomly selected 16 controls (9 males and 7 females; mean age 29.38 ± 3.20 years; range 15–60 years) who had no known neurological disease or exposure to AEDs. Controls were patients treated for increased intracranial pressure due to head trauma and had histologically normal temporal neocortical samples. All patients with TLE included in our study had typical clinical manifestations and characteristic EEGs of epilepsy (Table 1). Before the operation, a detailed medical history was collected, including the type of seizure, seizure frequency, AEDs, and patients underwent a complete physical examination. For identification of the epileptic foci in each patient, EEG recordings and neuroradiological testing were performed, including brain X-ray computerized (CT) scans and magnetic resonance imaging (MRI). We did not find any progressive lesions in the central nervous system as determined by cranial CT or MRI in the TLE group. All patients were resistant to at least three AEDs and experienced at least one seizure attack within 1 week before the surgery. Samples from these patients were taken only for treatment purposes. There was no significant difference in age or sex between the TLE patients and control subjects ( > 0.05).

### Mouse samples

All chronic epileptic mice brain tissues were from pilocarpine-induced epileptic mice with SRSs. Control mouse samples originated from animals injected with saline.

### Animals

For the pilocarpine and KA experiments, including the electrophysiology experiments, we used male adult C57BL/6 mice (20–30 g, 10–14 weeks old). The mice were housed five/cage under a 12-h light–dark cycle (lights on from 8 a.m. to 8 p.m.). All mice were housed under stable and specific-pathogen-free conditions (room temperature: 22 ± 1 °C; humidity: 55 ± 5%) with normal food pellets (Kaixieli Co., Ltd., Beijing, China) and fresh water provided ad libitum. All mice were all housed for 1 week for acclimatization before the experiments were performed.

### rAAV generation and stereotaxic injections

Packages of rAAVs (rAAV2/9) were provided by Bio-pharmaceutical Technology Co., Ltd. (Shanghai, China). AAV-EF1a-eGFP-U6-shRNA targeting sequences for to mouse ZDHHC8 (5ʹ-CTTCAGTATGGCTACCTTCAT-3ʹ) were tested for their ability to reduce cDNAs in co-transfected HEK293T cells^41^. These sequences were confirmed to effectively and specifically suppress ZDHHC8 expression. A neuron-specific promoter, the human synapsin I promoter (hSyn), was used to overexpress the ZDHHC8 gene (AAV-hSyn-EGFP-ZDHHC8). Its ability to increase ZDHHC8 expression in mice was verified in HEK293T cells. The ZDHHC8 genes were synthesized according to GenBank NM_172151.4 (Fig. S1A).

The control shRNA was a scrambled shRNA with an empty vector expressing GFP (rAAV-Scr-sh). The scrambled shRNA sequence was 5ʹ-TTCTCCGAACGTGTCACGT-3ʹ. An rAAV with empty vector expressing GFP alone (rAAV-Empty-GFP) was used as the control for the overexpression group. Viral titers were 3.6 × 10^12^ particles/ml for AAV-EF1a-eGFP-U6-shRNA (rAAV-ZDHHC8-sh), 6.58 × 10^12^ particles/ml for rAAV-Scr-sh, 3.75 × 10^12^ particles/ml for AAV-hSyn-eGFP-ZDHHC8 (rAAV-ZDHHC8), and 5.95 × 10^12^ particles/ml for rAAV-Empty-GFP.

In vivo viral infection was performed as described previously^17,21,42^. Adult mice were anesthetized with 3.5% chloral hydrate (100 g/ml) and placed in a stereotactic headframe (Reward Life Technology Co., Ltd., Shenzhen, China). Viral vectors were bilaterally targeted to the lateral ventricle (coordinates from the bregma: AP = −0.3 mm, *L* = −1.0 mm, and *V* = −2.0 mm below the dura). A microsyringe (Hamilton, Reno, NV) was filled with 2.0 µl of virus. The needle was inserted into the unilateral lateral ventricle, and 1.0 µl of virus was delivered over 10 min. After a 5-min delay, the needle was withdrawn by 0.25 mm, and an additional 1.0 µl of virus was delivered over an additional 10 min. The injection needle was withdrawn 5 min after the second infusion. Both rAAV-ZDHHC8-sh and rAAV-ZDHHC8 were localized in the CA1 and dentate gyrus of the hippocampus 3 weeks after ventricle (i.c.v.) injection (Fig. S1B). The expression of ZDHHC8 in the hippocampus of all mice was tested 1 week, 3 weeks, and 5 weeks after rAAV injections. We found that the mice were suitable for use 3 weeks after rAAV injections (Fig. S1C-S1D). Hippocampal protein levels of ZDHHC5, an unrelated family member, were not changed (Fig. S1C-S1D).

### In vivo chronic seizure model

#### KA-induced chronic seizure model

The KA-induced chronic epilepsy model was established as previously described^16–19^. The rAAV-treated mice were deeply anesthetized by 3.5% chloral hydrate (100 g/ml) and placed in a stereotactic headframe. A guide cannula (Plastics One) was placed into the right dorsal hippocampus CA1 area (coordinates from bregma: AP = −1.8 mm; *L* = −1.5 mm; and *D* = −1.5 mm below dura) for KA injection (0.3 μg in 50 nl 0.9% NaCl solution) (Sigma-Aldrich Co., St. Louis, USA). LFP recording depth electrodes, a 4 × 4 microwire array of platinum-iridium alloy wire, each with 25-μm diameter (Plexon, Dallas, TX), were implanted into the left dorsal hippocampus (coordinates from the bregma: AP = −1.8 mm, *L* = −1.5 mm, and *D* = −1.5 mm below the dura). After 1 week of postoperative recovery, KA was infused into the right hippocampus at a rate of 0.11 μl/min with a microsyringe. After infusion, the microsyringe was left in the right CA1 area for an additional 5 min. To minimize backflow along the injection trace, the microsyringe was slowly withdrawn from the brain over 2 min. To standardize the duration of seizure activity, we determined the time of behavioral SE onset when a mouse first reached a stage ≥4 seizure. A mouse that experienced a minimum of three stage 3–6 seizure events within 45 min following KA injection was considered to have undergone SE. Behavioral seizures were classified according to Racine’s scale^43^ for mice. To minimize mortality rates, diazepam (10 mg/kg, i.p.; Jinyao Amino Acid Co., Ltd., Tianjin, China) was administered 45 min after the onset of KA-induced SE, followed by lorazepam (6 mg/kg, i.p.; Jinyao Amino Acid Co., Ltd., Tianjin, China) 1 h later^12^. A total of 50 animals (40 experimental (rAAV-treated) and 10 non-treated age-matched controls) were used in our experiments. Approximately 5% of these mice experienced lethal seizures. Acute seizures were defined as seizures occurring within 24 h of KA injection^18^. Approximately 90% of these mice (*n* = 44) showed SRSs. Control mice corresponded to a non-injected sham operation group.

#### In vivo LFP multi-tetrode recordings from the CA1

After observation of SRSs (continuous observation for more than 30 days), we subsequently used mice that developed stable baseline of SRSs for in vivo LFP recordings using an OmniPlex® D neural Data Acquisition System (Plexon, Dallas, TX). LFP activity was continuously recorded and digitized at 4 kHz, filtered (0.1–1000 Hz) and preamplified (1000×) for 2 h per day for 7 days. Epileptiform-like discharge events were defined electrographically as high frequency (>5 Hz) and high amplitude (>2 times baseline) with a minimal duration of 5 s^16,19^. The duration of epileptiform activity was measured from the onset of the initial rise to the point when the LFP activity returned to baseline with no after-discharge period^44^. For each recording session, we analyzed the frequency (events/min) and duration of epileptiform-like discharges. NeuroExplorer® v4.0 (Plexon, Dallas, TX) was used for data analysis of epileptiform-like discharge events.

#### Pilocarpine-induced chronic seizure model

Chronically epileptic mice were obtained by the pilocarpine-induced SE model of TLE. All C57BL/6 mice received an intraperitoneal (i.p.) injection of pilocarpine (320 mg/kg; Sigma-Aldrich) 3 weeks after rAAV injections according to standard procedures^21,45^. To minimize the peripheral effects of cholinergic stimulation, the animals received methylscopolamine nitrate (0.1 mg/kg; Jinyao Amino Acid Co., Ltd., Tianjin, China) 30 min before pilocarpine administration. We determined the time of SE onset as the point when a mouse first reached a stage ≥4 seizure. To standardize the duration of seizure activity, a mouse that experienced continual recurrent seizures (stage 3–6 seizure) within 90 min following pilocarpine was considered to have undergone SE. It has been reported that more than 30 min of SE is necessary to trigger long-term modifications of neuronal circuits and chronic epileptogenesis. To minimize the mortality rates of the procedure, diazepam (10 mg/kg, i.p.) was administered to terminate seizures after 90 min^46^. A total of 70 mice (60 experimental (rAAV-treated) and 15 non-treated controls) were used in our experiments and all experienced SE. Approximately 10% of these mice experienced lethal seizures. All mice that experienced SE were observed for development of spontaneous seizures more than a month. Approximately 90% of these mice (*n* = 62) showed SRSs; the mice that did not develop spontaneous seizures were excluded from the analyses. Control mice corresponded to a non-injected sham operation group.

#### Spontaneous seizure assays of chronic models

During the first 24 h after SE induction, the mice were monitored to detect spontaneous seizures using a digital video-camera recording system. Seizures were scored by two blinded and experienced observers. The consistency in identifying SRSs between the observers was ~90%. When observers disagreed, the events were excluded from the study. Only seizures graded ≥3 on Racine’s scale^43^ (Stage 0, normal activity; Stage 1, staring, mouth and facial clonus; Stage 2, head nodding; Stage 3, monolateral forelimb clonus; Stage 4, rearing and bilateral forelimb clonus; Stage 5, bilateral forelimb clonus with rearing and falling; and Stage 6, generalized tonic-clonic seizure) were analyzed. Behaviors corresponding to Racine scores of 1–2 were deliberately excluded because the authors felt that the scoring of these minor behaviors would not be technically feasible due to subjectivity and would thus lead to substantial error^47^.

### Electrophysiology

#### Acute hippocampal slice preparation

Hippocampal slices from rAAV-treated and non-injected (control) C57BL/6 mice were prepared after 3 weeks. Briefly, the mice were anesthetized with 3.5% chloral hydrate (100 g/ml). After decapitation, the brain was rapidly removed and chilled in an ice-cold oxygenated cutting solution of ACSF containing the following (in mM): 92 choline chloride, 2.5 KCl, 1.2 NaH_2_PO_4_•H_2_O, 0.5 CaCl_2_•2H_2_O, 10 MgCl_2_•6H_2_O, 25 d-glucose, 30 NaHCO_3_, 20 HEPES, 5 Na-ascorbate, 3 Na-pyruvate, 2 thiourea, and 12 *N*-acetyl-cysteine at pH 7.4 (with 95% O_2_ and 5% CO_2_). Coronal hippocampal slices (400 μm) were transversely cut in cutting solution using a vibratome (VT1200S; Leica, Mannheim, Germany) and transferred to a storage chamber containing Mg^2+^-free ACSF that contained the following (in mM): 124 NaCl, 2.5 KCl, 1.25 NaH_2_PO_4_•2H_2_O, 26 NaHCO_3_, 0 MgCl_2_•6H_2_O, 2 CaCl_2_, and 10 glucose at pH 7.4 (with 95% O_2_, 5% CO_2_, and 300–315 mOsm/kg) at 34 °C for additional 1 h before recording.

#### In vitro seizure model

In this experiment, slices were transferred into a submerged recording chamber and continuously perfused using gravity feed at 2–3 ml/min with the oxygenated ACSF (95% O_2_, 5% CO_2_) at room temperature. Field potentials were recorded with a borosilicate glass microelectrode (1–2 MΩ) filled with 1 M NaCl positioned in the stratum radiatum of CA1 and were filtered at 1 kHz and amplified 1000× using the MultiClamp 700 B system (Molecular Devices, Palo Alto, CA) and digitized at 2 kHz with Digidata 1440 (Axon Instruments, USA). The glass microelectrode was pulled by a micropipette puller (P-97, Sutter instrument). The epileptiform activity was elicited by using continuously oxygenated Mg^2+^-free and high [K^+^] ACSF contained the following (in mM): 124 NaCl, 8.5 KCl, 1.25 NaH_2_PO_4_•2H_2_O, 26 NaHCO_3_, 0 MgCl_2_•6H_2_O, 2 CaCl_2_, and 10 glucose. Once the frequency of the paroxysmal activity was stable for at least 10 min, the epileptiform events were recorded for the following 30 min. The anticonvulsant effects were evaluated by measuring the change in the frequency and durations of the ictal and interictal-like discharges. The amplitude of spikes of epileptiform discharge events (peak-to-peak) was ~200 μV, and spikes approximately two times larger than the noise level were accepted^48^. The duration of an epileptiform event was measured as the time interval between the first and last spike present in each epileptiform event. Interictal-like events were defined as event durations <1 s, and ictal-like events were defined as events with durations greater than 2 s, as previously reported. Interictal-like epileptiform events occur more than 3 events/min, and ictal (seizure)-like epileptiform events occur <1 event/min, as described previously^49^.

#### Whole-cell recording in pyramidal neurons

The whole-cell recording was prepared as previously described^27,50,51^. After recovery, slices were placed in a recording chamber and continuously superfused (1.5–2 ml/min) with oxygenated Mg^2+^-free ACSF at room temperature. Whole-cell patch-clamp recordings were performed in CA1 pyramidal neurons using a Digidata 1440A, pCLAMP 10.0.3.1 and MultiClamp 700B amplifier. Pyramidal neurons were visually selected using an upright infrared (IR) Olympus (BX51WI) microscope (Olympus, Tokyo, Japan) with ×40 water-immersion objective lens (×40, NA: 0.8), an IR camera (Dage-MTI, Michigan City, IN, USA) and a video recording system. Pyramidal neurons have a typical soma (cell body) that is shaped like a teardrop or a rounded pyramid and one projecting axon to which dendrites bind in a thick band called the stratum radiatum^52^. For whole-cell recordings, pipettes were filled with patch solution with a resistance of 3–6 MΩ.

For AP recordings, pyramidal neurons were held at resting membrane potential in current clamp mode. The patch solution contained the following (in mM): 60 K_2_SO_4_, 60 NMG, 40 HEPES, 4 MgCl_2_•6H_2_O, 0.5 BAPTA, 12 Na-phosphocreatine, 2 Na-ATP, and 0.2 Na_3_-GTP. The epileptiform activity of pyramidal neurons was induced by application of Mg^2+^-free ACSF. Four or more APs were defined as a burst and quantified as a PDS driven by high frequency APs^24,53^.

For excitatory recordings, the patch solution contained the following (in mM):130 CsMeSO_4_, 10 HEPES, 10 CsCl, 4 NaCl, 1 MgCl_2_•6H_2_O, 1 EGTA, 12 Na-phosphocreatine, 0.5 Na_3_-GTP, 5 Mg-ATP, and 5 NMG. For inhibitory recordings (mIPSC), the patch solution contained (in mM): 100 CsCl, 10 HEPES, 1 MgCl_2_•6H_2_O, 1 EGTA, 30 NMG, 0.5 Na_3_-GTP, 5 Mg-ATP, 1 EGTA, and 12 Na-phosphocreatine (pH 7.2, 280–290 mM mOsm). For assessments of mEPSCs, voltage-clamp recordings were performed at a holding potential of −70 mV in the presence of the voltage-gated Na^+^ channel blocker tetrodotoxin (TTX, 1 μM) (Shanghai Aladdin Bio-Chem Technology Co., China) and the GABAA receptor blocker picrotoxin (PTX, 100 μM) (Sigma-Aldrich, USA). The mIPSCs were similarly recorded but in the presence of 20 μM DNQX (Sigma-Aldrich, USA), 50 μM AP5 (Sigma-Aldrich, USA) and 1 μM TTX. Cumulative probability plots were constructed using 200 events.

For AMPA/NMDA analysis, in the presence of 100 μM PTX, the AMPAR-mediated current responses were measured as the peak amplitude at −70 mV in response to stimulation of the Schaffer collaterals. Then, the membrane potential was voltage clamped at +40 mV, and an amplitude of 50 ms post-stimulus was identified as the NMDA receptor-mediated response. Ten sweeps were evoked through 0.05-Hz current pulses (0.1 ms duration) by stimulating the SC-CA1 pathway^54^ using tungsten microelectrodes (California FineWire Company, Grover Beach, CA) for each membrane potential. For PPR recordings, the holding potential was −70 mV in the presence of 100 μM PTX and 50 μM AP5. The interval for paired stimulations was set at 50 ms. PPR was defined as the ratio of the amplitude of the second synaptic response to the amplitude of the first synaptic response.

To measure inward rectification in evoked AMPA receptor-mediated responses, the tests were performed with 100 μM picrotoxin and 50 μM AP5 in ACSF; 100 µM spermine (Sigma-Aldrich, USA) was added to excitatory intracellular solutions. To generate *I*–*V* curves for rectification measurements, cells were held at ranges of −60, −40, −20, 0, +20, +40, and +60 mV. The absolute amplitude of average AMPAR-mediated current responses at −60 mV/+40 mV was defined as the RI. The values of AMPA-R EPSCs obtained were normalized at −60 mV as a function of holding potential (mV) to determine the inward rectification at positive holding potentials in ordinate^25^. In whole-cell recording experiments, series resistance was controlled below 20 MΩ and not compensated. Cells were rejected if series resistance fluctuated more than 25% of the initial. The recording lengths were more than 5 min after a stable recording.

### Western blotting

Western blotting was performed, and human brain tissues were prepared as previously described^29^. All mice were sacrificed after behavioral observation. The hippocampus and cortex were separated and stored in liquid nitrogen for subsequent use. Human lysates and hippocampal and cortex homogenates were subjected to SDS-polyacrylamide gel electrophoresis (SDS-PAGE), transferred to membranes, and probed with antibodies for the following: ZDHHC8, ZDHHC5, and GADPH. The immunoreactivity of individual bands on Western blots was measured by ImageJ software (National Institutes of Health) and normalized to GADPH immunoreactivity. Representative results of immunoblotting from at least three independent experiments are shown.

### Immunoprecipitation

Mice were anesthetized and decapitated, and brain tissue was quickly extracted. The hippocampus was rapidly dissected on ice, incubated in modified RIPA buffer (Beyotime, Nantong, China) on ice for 15 min, and centrifuged at ~200,000 × *g* for 10 min at 4 °C. The supernatant was collected and supplemented with 100 µl protein A/G-agarose beads overnight at 4 °C on a wheel. The beads were then allowed to sediment at the bottom of the tube, and the supernatant was collected. ZDHHC8 (1:200, Abcam), rabbit anti-GluA1 pAb (1:500; Abcam), rabbit anti-GluA2 pAb (1:200; Proteintech Group, Inc.), rabbit anti-GluA3 pAb (1:500; Bioworld Technology, Inc.), and rabbit anti-GluA4 pAb (1:200; Proteintech Group, Inc.) antibodies were incubated in equal amounts with the supernatant. Protein A/G-agarose beads were added to the supernatant, and the supernatant was incubated for 2 h at room temperature on a wheel. The beads were then washed three times with RIPA buffer and submitted to SDS-PAGE. The mixture was boiled for 10 min. The Western blots were detected using ZDHHC8 and GluA1, GluA2, GluA3, and GluA4 antibodies for immunoprecipitation^21^.

### Immunofluorescence

After epileptic and control mice were deeply anesthetized and the hearts were perfused with 4% paraformaldehyde (PFA), the brain tissue was removed. Then, the tissues were postfixed in 4% PFA at 4 ˚C for 8 h. The brains were dehydrated with 30% sucrose in PBS for ~48 h at 4 ˚C, and then covered in optimal cutting temperature compound (OCT, Tissue-Tek), frozen by slow immersion in chilled isopentane, cryoprotected, and cut into 30 µm coronal sections (−1.8 mm to −2.3 mm relative to bregma) on a cryostat (CM1860; Leica, Mannheim, Germany)^51^. The neocortical tissue from TLE patients (10 slides were cut from each sample, 16 samples from TLE patients and 10 samples from epileptic mice, and three slides were randomly selected from each sample for immunofluorescence) was removed from liquid nitrogen for the same dehydration treatment and then frozen in sections. Frozen sections were immersed in 100% acetone for 30 min at room temperature and then incubated with 1% Triton-X 30 min at 37 °C. Then, sections were blocked with 10% goat serum (Beijing Zhongshan Golden Bridge Co., Ltd.) for 30 min at 37 °C and finally washed with ice-cold PBS. Sections were then incubated with a mixture of polyclonal rabbit anti-ZDHHC8 with either guinea pig anti-MAP2 mAb, chicken anti-GAD67 mAb, mouse anti-GFAP mAb, mouse anti-PSD95 mAb, guinea pig anti-VGLUT1 mAb, mouse anti-Gephyrin mAb, or DAPI at 4 °C overnight. Sections were washed and incubated with Alexa Fluor 488-coupled goat anti-rabbit (1:50, Beyotime, Inc.), Alexa Fluor 594-coupled goat anti-mouse (1:50, Beyotime, Inc.), Alexa Fluor 647-labeled goat anti-guinea pig IgG (1:200, Beyotime, Inc.), or Alexa Fluor 405-labeled goat anti-chicken IgG (1:400, Abcam) in a darkroom for 60 min at 37 °C. They were then washed with PBS and mounted in 1:1 glycerol/PBS. Fluorescence images were captured via laser scanning confocal microscopy (Leica Microsystems) using an Olympus IX 70 inverted microscope (Olympus) equipped with a FluoView FVX confocal scan head.

The ZDHHC8-positive cells that were co-localized with MAP-2 and showed a visible nucleus and/or a complete cell contour were counted in the cerebral cortex and the CA1 and CA3 pyramidal cell layers of the dorsal hippocampus in the epileptic brains vs. control brains (*n* = 8 in each group). Cell counting in humans was performed by evaluating the temporal cortex regions (*n* = 8 in each group). In each sample, two representative adjacent non-overlapping fields of the areas of interest were captured using rectangular frames (magnification ×40; 300 µm × 300 µm) and digitized using a laser scanning confocal microscope.All cell types within the captured images were marked by one operator blinded to the treatment conditions, and an automated cell count was generated. The data obtained in each hippocampal subfield were added together to provide one single value per slice in each sample. Although this cell counting method has some limitations compared to designed-based stereological analyses, the occurrence of any bias in counting should similarly affect the control and experimental samples since these samples underwent the same procedures in parallel.

### Antibodies

The following primary antibodies were used in this study: purified rabbit anti-ZDHHC8 pAb (1:200; Abcam); rabbit anti-ZDHHC8 pAb (1:100; Bioss); rabbit anti-ZDHHC5 pAb (1:500; Proteintech Group, Inc.); rabbit anti-GADPH mAb (1:1000; Proteintech Group, Inc.); guinea pig anti-MAP2 mAb (1:200; Synaptic Systems); mouse anti-GFAP mAb (1:100; Proteintech Group, Inc.); chicken anti-GAD67 mAb (1:300; Synaptic Systems); mouse anti-PSD95 mAb (1:50; Proteintech Group, Inc.); guinea pig anti-VGLUT1 mAb (1:200; Synaptic Systems); mouse anti-gephyrin mAb (1:50; Santa Cruz Biotechnology); DAPI (1:50; Sigma-Aldrich); rabbit anti-GluA1 pAb (1:500; Abcam); rabbit anti-GluA2 pAb (1:200; Proteintech Group, Inc.); rabbit anti-GluA3 pAb (1:500; Bioworld Technology, Inc.); rabbit anti-GluA4 pAb (1:200; Proteintech Group, Inc.); Anti-Sodium Potassium ATPase mAb (1:1000; Abcam); and GABAAR β2/3 (1:200; Proteintech Group, Inc.).

### HE staining

After 3 weeks, rAAV-injected and non-injected C57BL/6 male mice were anesthetized with 3.5% chloral hydrate (100 g/ml) and intracardially perfused with 0.9% saline, followed by 4% PFA in 0.1 M phosphate buffer (pH 7.4). Subsequently, the brain was removed and postfixed in 4% PFA at 4 ˚C overnight and sectioned into frontal sections for histological analyses. Coronal sections from the dorsal hippocampus (−1.8 mm to −2.3 mm relative to bregma) were analyzed^55^. The brain regions mainly included the hippocampal CA1 and CA3 pyramidal cell layers and the dentate gyrus (*n* = 5 per group).

HE staining was performed in hippocampal tissue samples to detect neuronal loss. First, 4-µm-thick sections were deparaffinized, hydrated, stained with hematoxylin for 5 min, and washed under low speed running tap water for 2 min. Then, sections were counter-stained with eosin for 10 s and washed again. Slides were then dehydrated and cover-slipped. Images were obtained at ×4 by a light microscope (Olympus, Japan).

### Statistical analysis

The data were analyzed using Clampfit 10.0.3 (Molecular Devices), Mini Analysis Program software (version 6.0.3; Synaptosoft, Decatur, GA, USA), GraphPad 6 (GraphPad software, La Jolla, California, USA), SPSS 19.0 (SPSS Inc., Chicago, IL, USA), and CorelDRWX4 (Corel, Canada). The values are presented as the mean ± SEM. Unless otherwise noted, comparisons between two groups were analyzed using unpaired Student’s *t*-tests, comparisons of >2 groups were performed by one-way ANOVA, and multi-group comparisons were analyzed by two-way ANOVA followed by Bonferroni post hoc tests. For cumulative probability plots, Kolmogorov–Smirnov (KS) tests were used. *P* < 0.05 was considered statistically significant.

## Electronic supplementary material

Figure S1

Figure S2

Figure S3

Figure S4

Figure S5

Figure S6

Figure S7

Supplementary material
