# Supplementary figures and images for: ZDHHC8 critically regulates seizure susceptibility in epilepsy
Source: Cell Death Dis. 2018 Jul 23;9(8):795. doi: 10.1038/s41419-018-0842-0 (PMC6056564; doi:10.1038/s41419-018-0842-0)

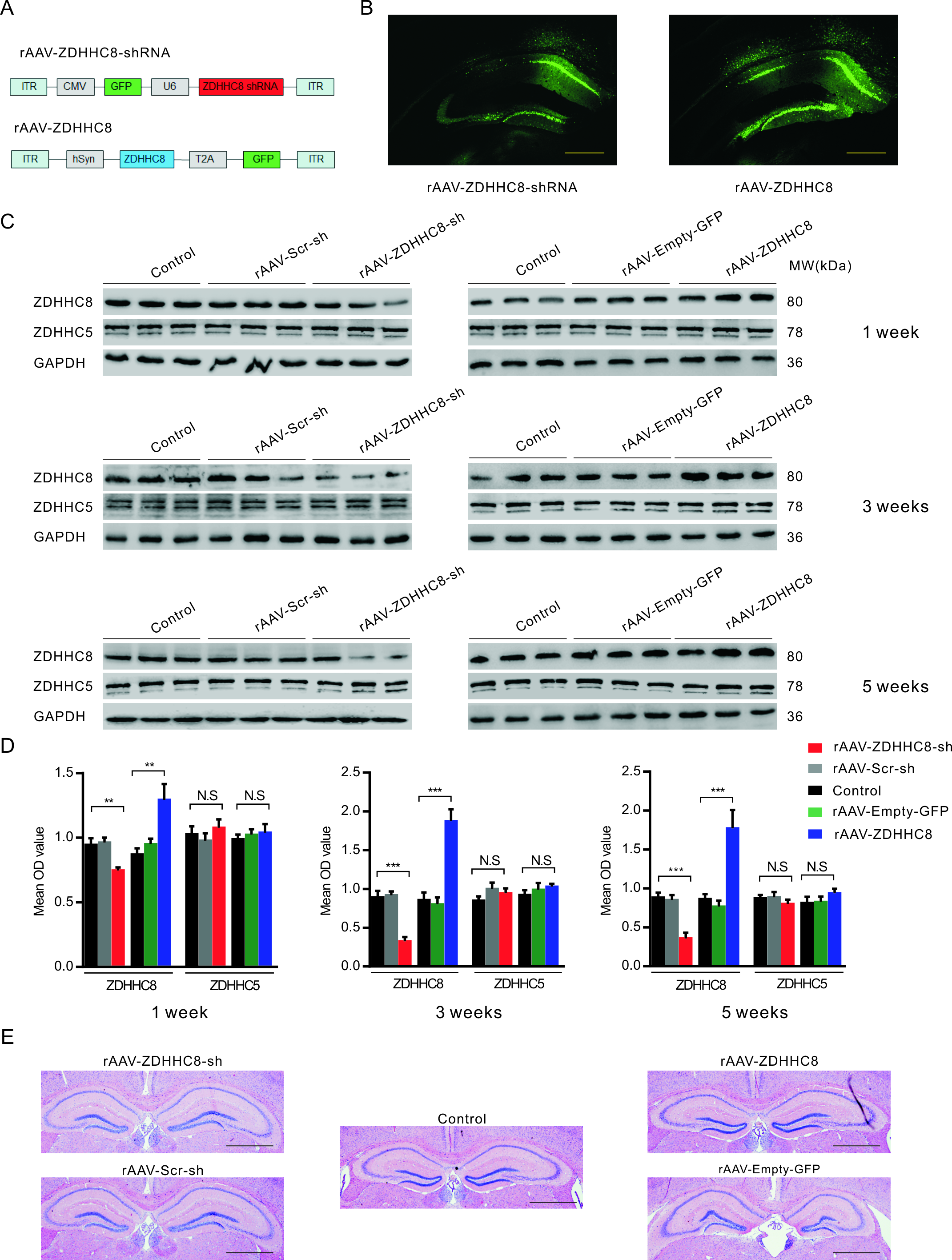

Supplement: Supplementary file 1 — Figure S1 [file 41419_2018_842_MOESM1_ESM.tif]

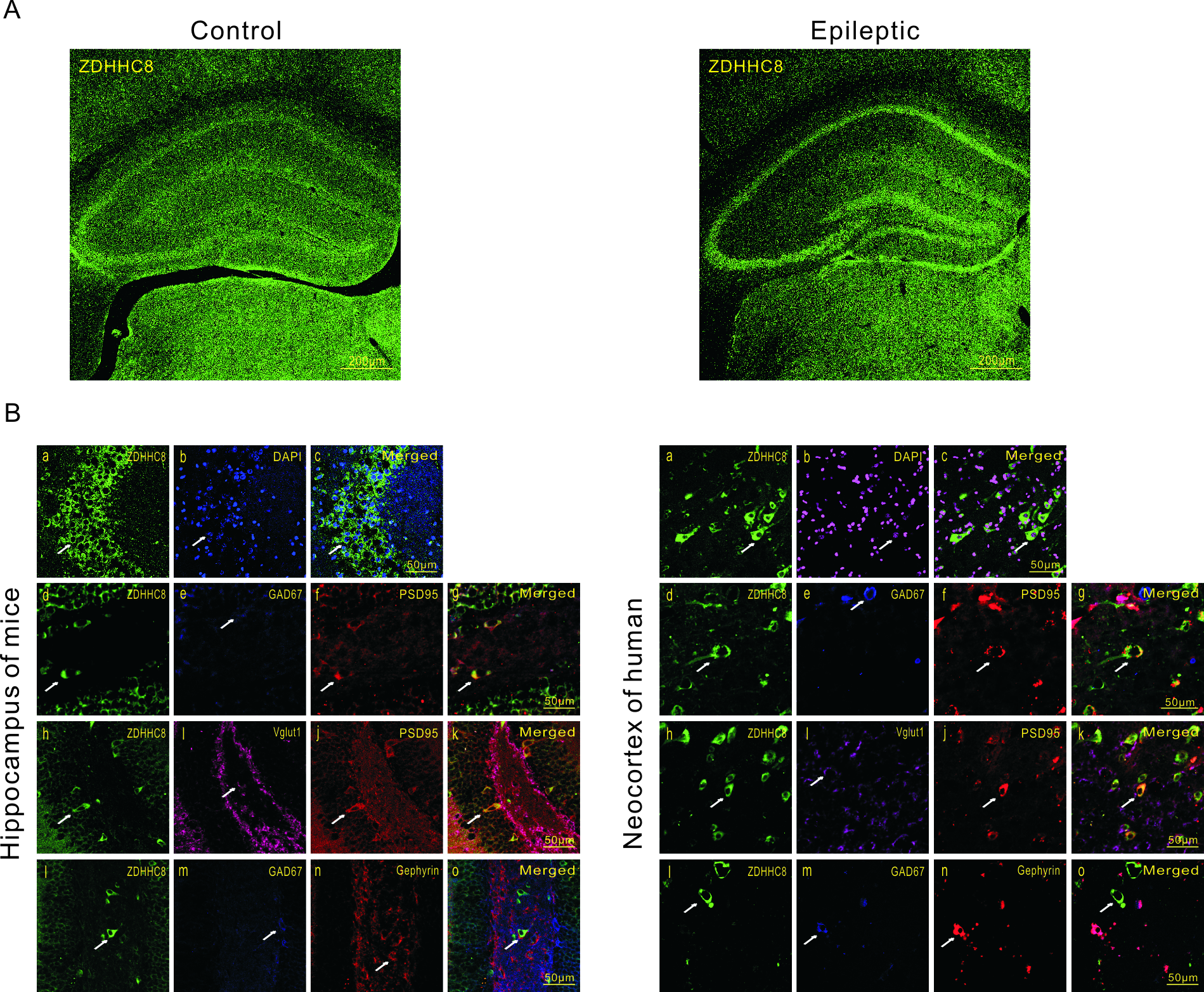

Supplement: Supplementary file 2 — Figure S2 [file 41419_2018_842_MOESM2_ESM.tif]

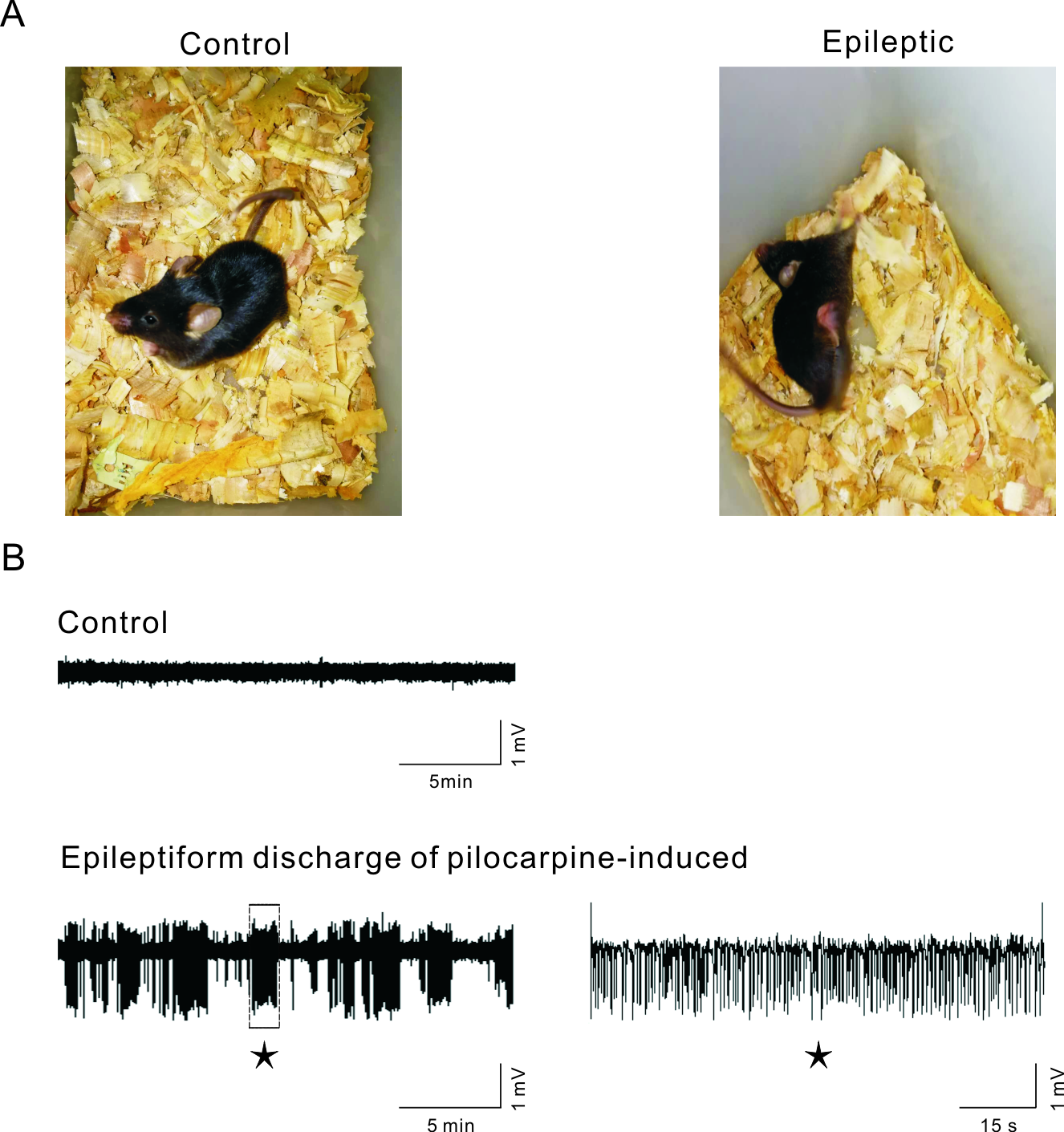

Supplement: Supplementary file 3 — Figure S3 [file 41419_2018_842_MOESM3_ESM.tif]

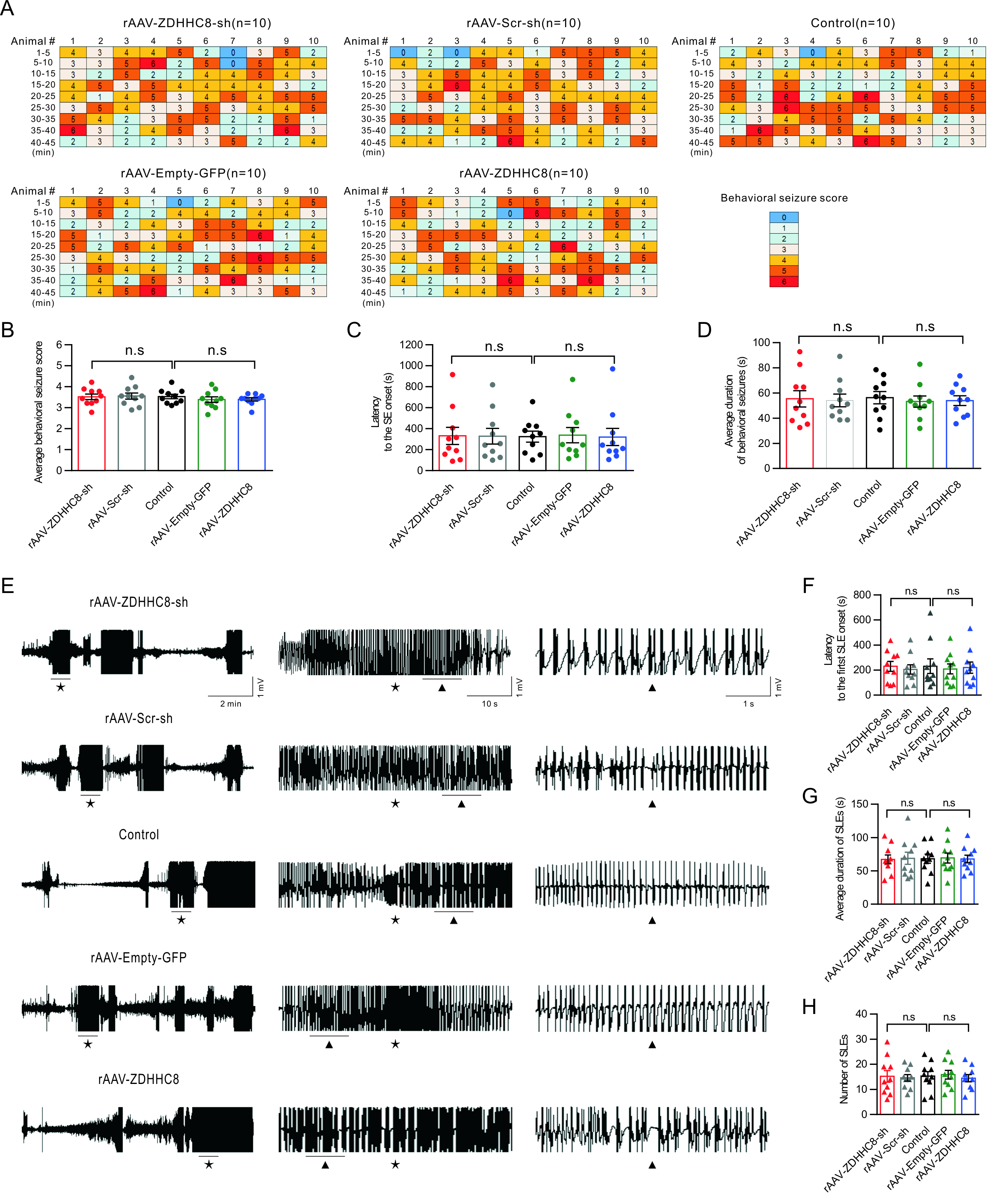

Supplement: Supplementary file 4 — Figure S4 [file 41419_2018_842_MOESM4_ESM.tif]

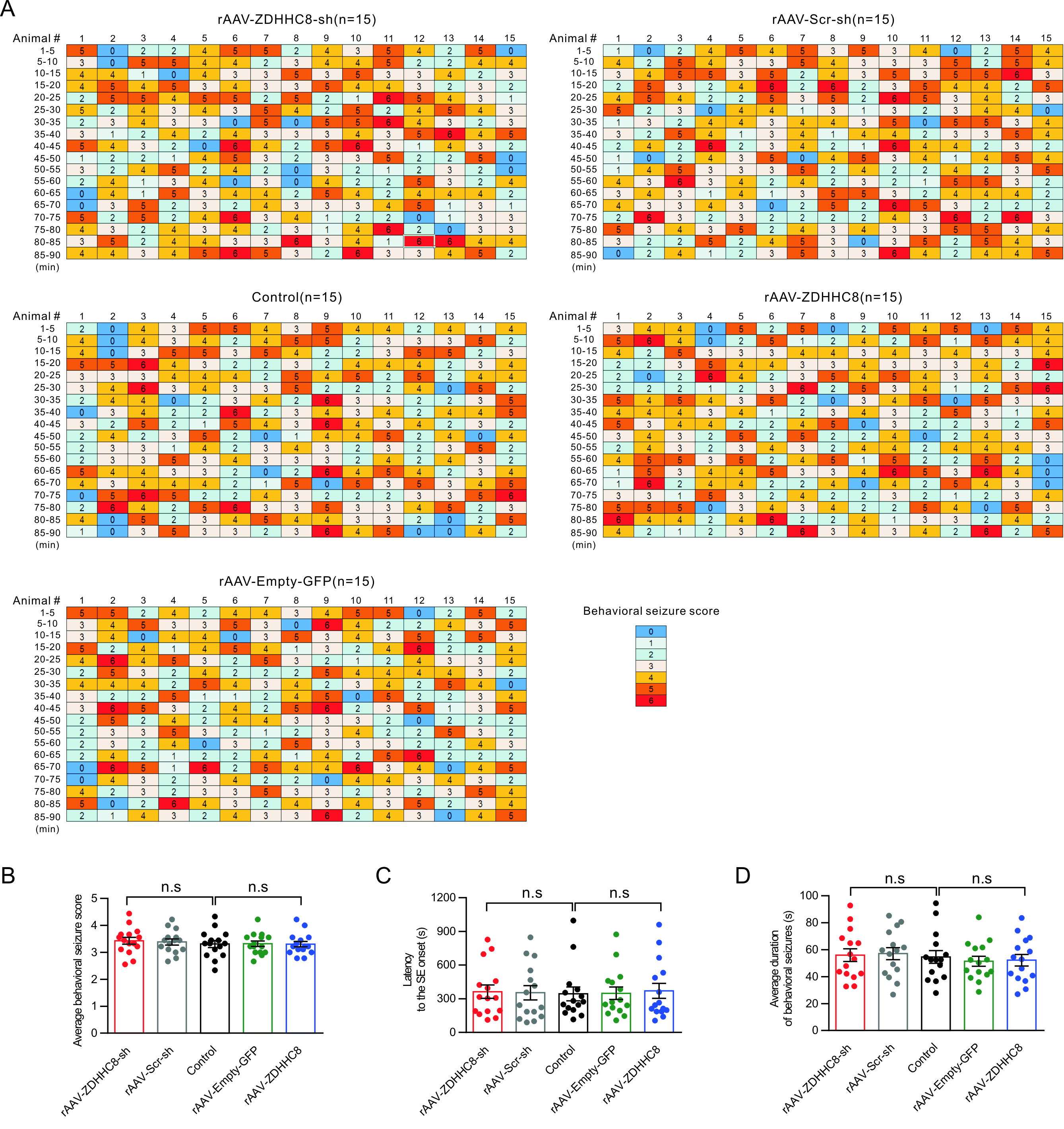

Supplement: Supplementary file 5 — Figure S5 [file 41419_2018_842_MOESM5_ESM.tif]

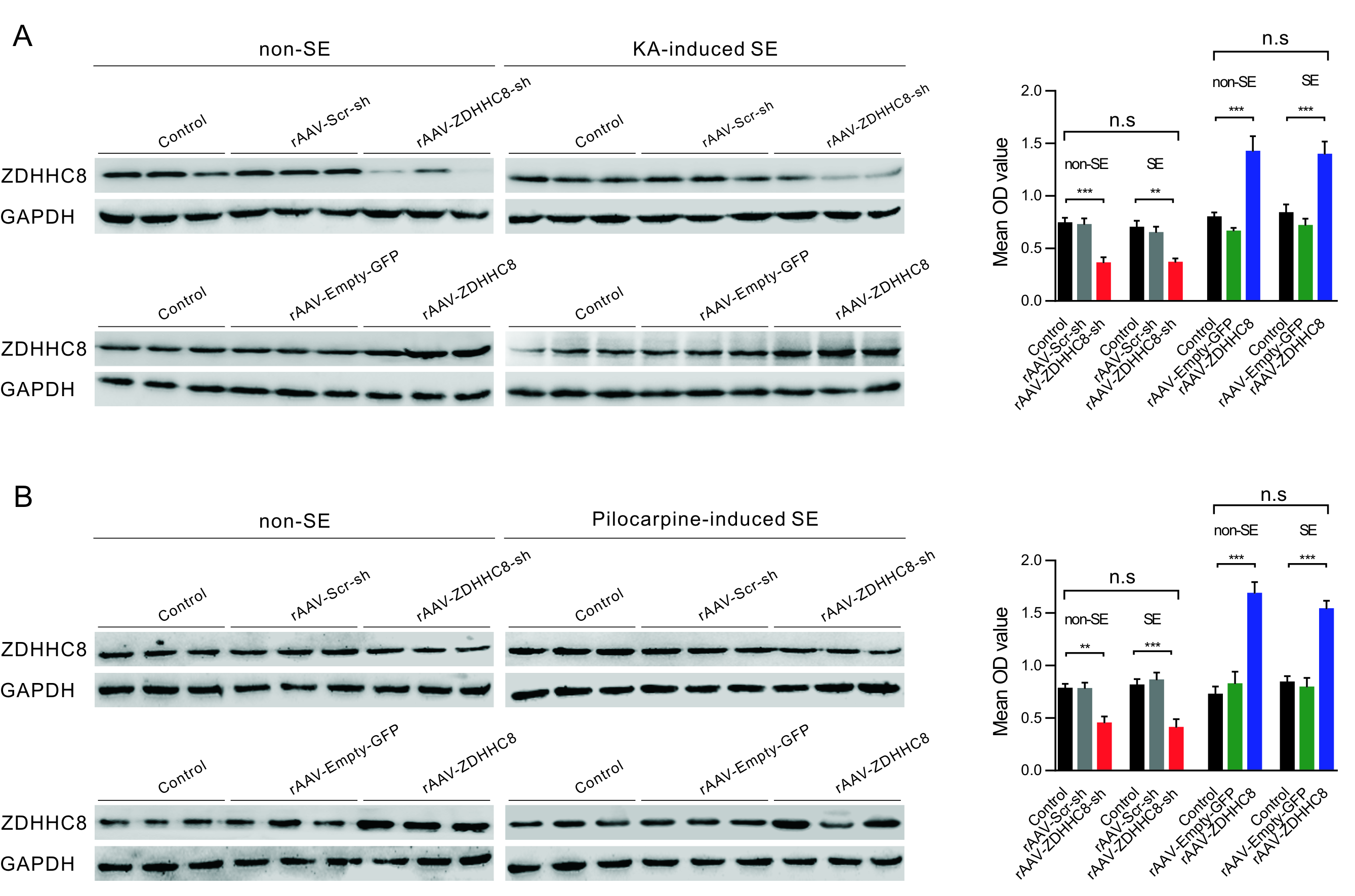

Supplement: Supplementary file 6 — Figure S6 [file 41419_2018_842_MOESM6_ESM.tif]

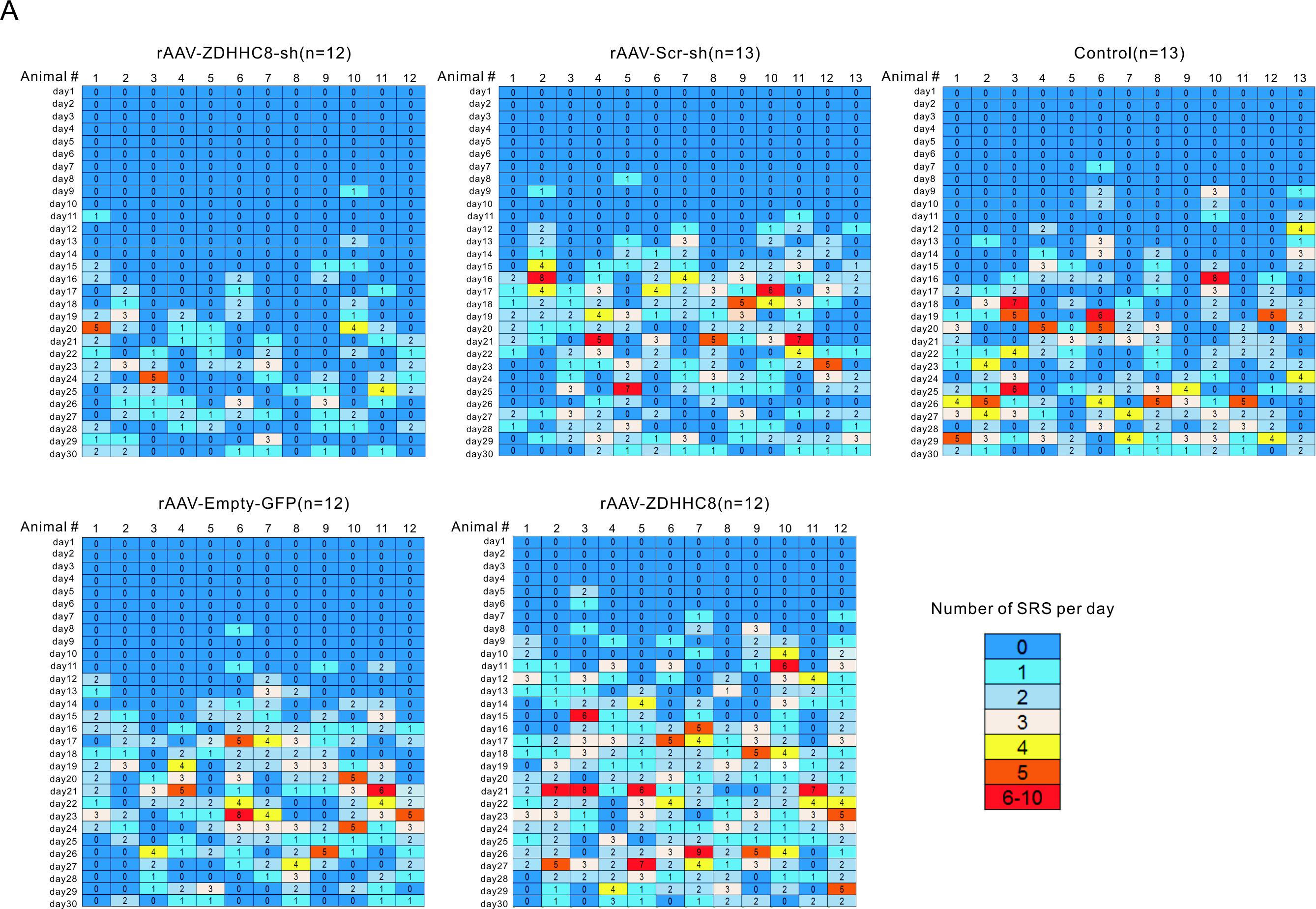

Supplement: Supplementary file 7 — Figure S7 [file 41419_2018_842_MOESM7_ESM.tif]
