# Supplementary material for: ZDHHC8 critically regulates seizure susceptibility in epilepsy
Source: Cell Death Dis. 2018 Jul 23;9(8):795. doi: 10.1038/s41419-018-0842-0 (PMC6056564; doi:10.1038/s41419-018-0842-0)
Supplement: Supplementary file 8 — Supplementary material [file 41419_2018_842_MOESM8_ESM.docx]

**Supplementary material**

**Materials and Methods**

**1. KA-induced status epilepticus**

The KA-induced chronic epilepsy model was established as previously described [^1-4^](#_ENREF_1). The rAAV-treated mice were deeply anesthetized by 3.5% chloral hydrate (100 g/ml) and placed in a stereotactic headframe. A guide cannula (Plastics One) was placed into the right dorsal hippocampus CA1 area (coordinates from bregma: AP=-1.8 mm; L=-1.5 mm; and D=-1.5 mm below dura) for KA injection (0.3 μg in 50 nl 0.9% NaCl solution) (Sigma-Aldrich Co., St. Louis, USA). LFP recording depth electrodes, a 4 × 4 microwire array of platinum-iridium alloy wire, each with 25-μm diameter (Plexon, Dallas, TX), were implanted into the left dorsal hippocampus (coordinates from the bregma: AP=-1.8 mm, L=-1.5 mm, and D=-1.5 mm below the dura). After one week of postoperative recovery, KA was infused into the right hippocampus at a rate of 0.11 μl/min with a microsyringe. After infusion, the microsyringe was left in the right CA1 area for an additional 5 min. To minimize backflow along the injection trace, the microsyringe was slowly withdrawn from the brain over 2 min. After KA injection, we recorded LFP activity and performed time-locked video-monitoring in the mice to assess SE for 45 min. Monitoring started 20 min before hippocampal KA infusion for recording the baseline LFP and behavioral activity. To standardize the duration of seizure activity, we determined the time of behavioral SE onset when a mouse first reached a stage ≥4 seizure. A mouse that experienced a minimum of three stage 3–6 seizure events within 45 min following KA injection was considered to have undergone SE. Behavioral seizures were classified according to Racine’s scale [^5^](#_ENREF_5) for mice. The LFP duration of SE was deﬁned as the time between the occurrence of the ﬁrst seizure-like event (epileptiform discharges) in the LFP and the time point after which no more high-frequency, high-amplitude discharges were observed erved erved, which should include discrete electrographic seizures, waxing and waning epileptiform activity, continuous, high amplitude, rapid spikings or periodic epileptiform discharges on a relatively flat background[^1^](#_ENREF_1)^,^[^6^](#_ENREF_6).

**Supplementary Figure Legends**

**Figure S1: Analysis of the differential expression of ZDHHC8 after rAAV injection.**

(**A**) Schematics of rAAV ZDHHC8 shRNA and ZDHHC8-overexpression vectors. ITR, inverted terminal repeats; CMV, cytomegalovirus promoter; hSyn, human synapsin I promoter; T2A, self-cleaving peptide. (**B**) Immunoﬂuorescence staining showing rAAV-ZDHHC8-shRNA and rAAV-ZDHHC8 expression in the CA1 and DG 3 weeks after injection; scale bar, 100 µm. (**C-D**) Western blot images showing ZDHHC8, ZDHHC5 protein levels in mice after rAAV-ZDHHC8-sh, rAAV-Scr-sh, rAAV-Empty-GFP, and rAAV-ZDHHC8 injection at weeks 1, 3, and 5. The data represent the mean±SEM. Statistical signiﬁcance was evaluated by one-way ANOVA (n=6 per group, **P<0.01, ***P<0.001). **(E)** Effects of rAAV injection on neuronal tissues at 3 weeks. HE staining of hippocampal sections (n=5; scale bar, 100 µm).

**Figure S2: ZDHHC8 immunoreactivity in the hippocampus and neocortex.**

(**A**) The location of ZDHHC8 in the hippocampus of epileptic and control mice; scale bar, 200 µm. (**B**) The immunoreactivity of ZDHHC8 in the hippocampus of control mice and in the human temporal neocortex of the control group. (**a-c**) ZDHHC8 expression (green) in the cell membrane. (**d-g**) ZDHHC8 expression (green) and PSD95 (red) were co-localized, but no co-localization was observed with GAD67. (**h-k**) ZDHHC8 (green) and VGLUT1 (purple) were not co-localized, but co-expression with PSD95 was observed. (**l-o**) ZDHHC8 (green), GAD67 (blue) and Gephyrin (red) were not colocalized, indicating that ZDHHC8 was not located in inhibitory synapses; scale bar, 50 μm. The white arrows in all the images indicate positive neurons.

**Figure S3: Behavioral seizure and LFP recording in the pilocarpine-induced chronic seizure model. (A)** Photography showing a mouse with normal activity (left) and a spontaneous generalized tonic-clonic seizure in a mouse (right). **(B)** Representative epileptiform-like discharge events with LFP recording in a mouse with spontaneous recurrent seizures (SRSs). The pentagram represents the expansions of the tracings of epileptiform-like discharge events.

**Figure S4: Equivalent severity of SE is induced by KA infusion following ZDHHC8-related rAAV treatment in mice. (A)** Representative heat map showing the behavioral seizure severity during 45 min of KA-induced SE in rAAV-treated mice. The maximum seizure score of each animal was measured every 5 min following infusion of KA. **(B)** Average behavioral seizure scores, **(C)** Latency to the SE onset and **(D)** Average duration of behavioral seizure represented no significant difference (n.s.) between control and rAAV-treated mice by one-way ANOVA test (n=10). **(E)** Representative LFP tracings were recorded during 45 min of from KA-induced SE mice. Pentagrams and triangles represent the expansions of the tracings of seizure-like (epileptiform-like) events (SLEs). **(F)** Latency to the first SLEs onset, **(G)** Average duration of SLEs and **(H)** The number of SLEs showed no significant differences between each group by one-way ANOVA test (n=10). Data are presented as mean±SEM.

**Figure S5: Equivalent severity of SE is induced by pilocarpine infusion following ZDHHC8-related rAAV treatment in mice. (A)** Representative heat map showing the behavioral seizure severity during 90 min of pilocarpine-induced SE in rAAV-treated mice. The maximum seizure score of each animal was measured every 5 min following infusion of pilocarpine. **(B)** Average behavioral seizure scores, **(C)** Latency to the SE onset and **(D)** Average duration of behavioral seizure represented no significant differences (n.s.) between control and rAAV-treated mice by one-way ANOVA test (n=15). Data are presented as mean±SEM.

**Figure S6: ZDHHC8 expression following SE in KA and pilocarpine-induced seizure models. (A, B)** Left, representative Western blots of hippocampal lysates treated with rAAV-ZDHHC8-sh, rAAV-Scr-sh, rAAV-Empty-GFP and rAAV-ZDHHC8 3 weeks after completion of KA **(A)** and pilocarpine-induced SE **(B)**. Right, quantitative analyses of Western blots shows no difference in each group after SE compared with non-SE. The non-SE animals underwent treatment with vehicle but not chemoconvulsant. The data represent the mean±SEM. Statistical signiﬁcance was evaluated by two-way ANOVA with Bonferroni post hoc test, n=6 for each group. n.s. = not significant. **p<0.01, ***p<0.001.

**Figure S7: SRSs detected in the pilocarpine-induced chronic seizure model. (A)** Heatmap showing the number of SRSs of ZDHHC8-related rAAV-treated mice detected each day during days 1-30 after SE induction in the pilocarpine-induced seizure model (n = 12-13). The time-scale in each box is one day, one mouse per column.

**References**

1 Gu, B. *et al.* A Peptide Uncoupling BDNF Receptor TrkB from Phospholipase Cgamma1 Prevents Epilepsy Induced by Status Epilepticus. *Neuron* **88**, 484-491, (2015).

2 Jimenez-Mateos, E. M. *et al.* Silencing microRNA-134 produces neuroprotective and prolonged seizure-suppressive effects. *Nature medicine* **18**, 1087-1094, (2012).

3 Carriero, G. *et al.* A guinea pig model of mesial temporal lobe epilepsy following nonconvulsive status epilepticus induced by unilateral intrahippocampal injection of kainic acid. *Epilepsia* **53**, 1917-1927, (2012).

4 Maroso, M. *et al.* Toll-like receptor 4 and high-mobility group box-1 are involved in ictogenesis and can be targeted to reduce seizures. *Nature medicine* **16**, 413-419, (2010).

5 Racine, R. J. Modification of seizure activity by electrical stimulation. II. Motor seizure. *Electroencephalography and clinical neurophysiology* **32**, 281-294, (1972).

6 Raedt, R. *et al.* Seizures in the intrahippocampal kainic acid epilepsy model: characterization using long-term video-EEG monitoring in the rat. *Acta neurologica Scandinavica* **119**, 293-303, (2009).
